# Endothelial-to-mesenchymal transition and vasculogenic mimicry in tumor vascular plasticity: a comparative bibliometric analysis and mechanistic framework

**DOI:** 10.3389/fonc.2026.1944544

**Published:** 2026-09-25

**Authors:** Congcheng Sun, Pan Guo, Yiduo Fang, Hongyu Hu, Junqi Wang, Siqi Wang, Yuhang Wei, Chen Zhu

**Affiliations:** 1 Department of Neurosurgery, The First Hospital of China Medical University, Shenyang, Liaoning, China; 2 The Fourth Affiliated Hospital, China Medical University, Shenyang, Liaoning, China; 3 First Clinical Medical College, Shenyang Medical College, Shenyang, Liaoning, China; 4 The Department of Radiation Oncology, The First Hospital of China Medical University, Shenyang, Liaoning, China

**Keywords:** bibliometrics, endothelial-to-mesenchymal transition, tumor microenvironment, vascular plasticity, vasculogenic mimicry

## Abstract

**Background:**

Endothelial-to-mesenchymal transition (EndMT) and vasculogenic mimicry (VM) are distinct forms of cellular and vascular plasticity that may contribute to tumor vascular adaptation. Although they have largely evolved as separate research fields, emerging experimental observations suggest potential convergence in their regulation by the tumor microenvironment.

**Methods:**

We performed a comparative bibliometric analysis of EndMT and VM using the Web of Science Core Collection and Scopus for publications from 2005 to 2025. After cross-database deduplication and retraction screening using a custom open-source data-fusion pipeline, 4,746 records were retained (2,056 EndMT; 2,695 VM).Publication trends, collaboration networks, keyword co-occurrence, thematic structures, and citation bursts were analyzed using bibliometrix/Biblioshiny, VOSviewer, and CiteSpace.

**Results:**

Only five publications explicitly addressed both EndMT and VM in an oncological context, indicating limited formal overlap between the two literatures. Nevertheless, keyword co-occurrence, citation-burst, and thematic analyses revealed convergent research interests involving the tumor microenvironment, cellular plasticity, extracellular-matrix remodeling, and therapeutic resistance. These patterns motivated a mechanistic synthesis centered on three potential points of convergence: shared upstream signaling involving TGF-β, hypoxia/HIF-1α, and context-dependent Notch activity; EndMT-associated stromal and extracellular-matrix remodeling that may facilitate VM; and partial or hybrid cellular states.

**Conclusions:**

EndMT and VM should be considered distinct but potentially interconnected components of tumor vascular plasticity rather than simple alternatives to conventional angiogenesis. The bibliometric convergence provides a rationale for investigating their potential interactions, but does not establish a causal or bidirectional relationship. We therefore propose a testable conceptual framework in which shared microenvironmental pressures may influence both processes, while EndMT-associated stromal remodeling may provide one potential route toward a VM-permissive niche. Spatial transcriptomics, lineage tracing, co-culture models, and organoid systems may help determine whether these interactions occur *in vivo* and whether reciprocal regulation exists.

## 1 Introduction

Tumor progression depends on a dynamic vascular ecosystem in which endothelial cells, tumor cells, stromal cells, and immune cells interact to support growth, invasion, metastasis, and adaptation to therapeutic stress (1, 2). Tumor vascularization is not restricted to conventional VEGF-dependent sprouting angiogenesis. It involves a heterogeneous set of processes, including endothelial activation and state transitions, vessel remodeling and maturation, vessel co-option, vascular specialization, and adaptation of larger microvascular and arteriolar networks (1, 3). These programs can coexist within the same tumor and may vary according to tumor type, anatomical location, oxygen availability, and treatment pressure. Within this broader landscape of tumor vascular plasticity, EndMT and VM represent two distinct forms of cellular adaptation. EndMT involves a shift of endothelial cells toward mesenchymal-like states, whereas VM refers to the ability of aggressive tumor cells to form vessel-like, extracellular-matrix-rich channels without conventional endothelial lining (4–6). Rather than representing simple alternatives to angiogenesis, both processes may contribute to vascular adaptation under specific microenvironmental conditions (5–8). 

EndMT does not necessarily represent a complete loss of endothelial identity. Increasing evidence supports a spectrum of endothelial responses ranging from activation and dysfunction to partial or hybrid EndMT states and, in some contexts, more stable mesenchymal phenotypes (4, 9). Endothelial cells undergoing partial EndMT may retain endothelial features while acquiring selected mesenchymal characteristics, allowing them to remain functionally plastic within the tumor microenvironment. Accordingly, commonly used markers such as α-SMA, FSP-1, and vimentin, or the loss of CD31 and VE-cadherin, should not be interpreted in isolation as evidence of complete lineage conversion. VM represents a distinct form of tumor-cell plasticity. Tumor cells can acquire selected endothelial-associated programs, including VE-cadherin and EphA2, and organize into vessel-like channels that are commonly characterized histologically by periodic acid–Schiff (PAS)-positive extracellular matrix patterns (5, 6). PAS positivity reflects the presence of carbohydrate-rich extracellular components, including glycoproteins and basement-membrane-associated material, within these patterned structures. Importantly, PAS-positive channels should not be equated with conventional endothelial-lined blood vessels. Unlike EndMT, which involves phenotypic plasticity of endothelial cells, VM involves the acquisition of vessel-like properties by tumor cells. The cellular origins and immediate phenotypic programs of VM therefore differ from those of EndMT, although both may be influenced by overlapping microenvironmental signals. Their potential relationship remains incompletely understood.

The clinical relevance of vascular plasticity becomes particularly apparent in the context of anti-angiogenic therapy. Anti-angiogenic therapies, including the VEGF-targeting antibody bevacizumab and the multi-target tyrosine kinase inhibitor sunitinib, are widely used in cancer treatment, yet durable responses remain difficult to achieve in many solid tumors (7, 10, 11). Resistance can arise through multiple, context-dependent mechanisms, including compensatory activation of alternative pro-angiogenic pathways such as FGF, angiopoietins, and PDGF, vascular remodeling and co-option, stromal adaptation, and changes in endothelial or tumor-cell states (8, 11, 12). VM may represent another route of vascular adaptation in selected tumor contexts, particularly when hypoxia and treatment pressure favor tumor-cell plasticity (8, 13, 14). EndMT may likewise contribute to stromal remodeling and endothelial plasticity under specific microenvironmental conditions (4, 15, 16). Rather than viewing these processes as a linear sequence in which conventional endothelial vessels are replaced by VM, it may be more appropriate to consider EndMT and VM as potential components of a broader adaptive response to vascular stress.

Vasculogenic mimicry (VM) was first reported by Maniotis and colleagues in 1999 in aggressive melanoma. Since then, it has been observed in a wide range of solid tumors, including hepatocellular carcinoma, gastric and colorectal cancers, lung cancer, and breast cancer (5, 6). Pan-cancer analyses connect VM with several major oncogenic programs, most notably epithelial–mesenchymal transition (EMT), hypoxia-induced HIF-1α activation, and signaling through WNT/β-catenin and NOTCH pathways (17, 18). The clinical relevance of VM is increasingly recognized. In non-small cell lung cancer (NSCLC), VM is present in roughly 13.6%–45.2% of cases and serves as an independent marker of poorer survival (19). Broader meta-analyses across tumor types show a similar pattern, with VM consistently associated with reduced overall survival (20, 21). VM has also been linked to adjuvant therapy resistance and a higher likelihood of recurrence after surgery (22). Yet despite increasing recognition of its clinical relevance, the upstream microenvironmental cues that enable tumor cells to acquire the plasticity required for VM remain only partly understood. Recent findings point to a possible contribution of the endothelial compartment to this process. Through EndMT-associated changes in the tumor microenvironment, endothelial cells may help shape conditions that influence VM competence in neighboring tumor cells, although this possibility has received comparatively limited attention (15). 

Endothelial-to-mesenchymal transition (EndMT) was first described in cardiac development and later recognized as a driver of fibrosis and tumor stromal remodeling (23). EndMT involves the acquisition of mesenchymal characteristics by endothelial cells (4). During this process, endothelial cells may downregulate lineage-specific markers while acquiring mesenchymal traits. They become more motile, produce more extracellular matrix, and reshape the surrounding stroma. EndMT does not unfold as a simple switch. It usually progresses along a reversible continuum. Many cells settle into intermediate states where endothelial and mesenchymal markers are expressed together. These partial transitions can persist within the tumor microenvironment and add to its heterogeneity and plasticity (24). In tumors, lineage-tracing studies have provided evidence that endothelial-lineage cells can contribute to CAF-like populations in selected tumor contexts, including melanoma and pancreatic cancer (9). Endothelial-lineage cells with EndMT-associated phenotypes may contribute to the desmoplastic reaction characteristic of these tumors (9). They also increase the expression of matrix metalloproteinases and extracellular matrix components, changes that promote invasion (15, 25). Several signaling and microenvironmental pathways are relevant to both processes. TGF-β signaling, hypoxia-driven HIF-1α activity, and context-dependent Notch signaling have each been implicated in EndMT and VM, although their functions and downstream effects vary according to cell type and biological context (15, 26). This convergence raises the possibility that EndMT and VM may be influenced by overlapping regulatory environments rather than representing completely independent processes. Whether such convergence translates into direct functional interaction between the two processes remains unclear.

These observations raise the possibility that EndMT and VM may interact indirectly through the tumor microenvironment. One plausible route is that EndMT-associated stromal remodeling alters extracellular-matrix composition, protease activity, and paracrine signaling in ways that favor VM competence in neighboring tumor cells (9, 27). The reverse direction is less well established. VM-competent tumor cells may influence endothelial behavior through paracrine or contact-dependent signals (17, 28), but direct evidence demonstrating that VM induces EndMT remains limited. We therefore view a reciprocal relationship as a testable hypothesis rather than an established mechanism.

Despite the biological logic behind a possible EndMT–VM connection, this overlap has received relatively little direct attention. Most studies still treat the two processes separately, and only a small number of papers address them together within the same tumor setting. Research activity in both areas has grown over the past two decades, but these literatures have largely advanced in parallel. Cross-citation is limited, and efforts that attempt to synthesize both bodies of work remain rare. There are mechanistic reviews and prognostic meta-analyses for each process on its own (4, 5, 20, 21, 29). What is missing is a systematic comparative mapping of the research landscapes of EndMT and VM, including their thematic evolution, collaboration structures, citation patterns, and areas of overlap.

To address this gap, we performed a comparative bibliometric analysis using literature retrieved from both Web of Science and Scopus. We used an integrated retrieval strategy and processed the combined dataset through a custom, open-source fusion pipeline that performs automated deduplication, retraction checks, and refined search filtering. This workflow helps minimize the coverage bias that can arise from single-database studies. The analytical approach considered EndMT and VM both individually and at their point of overlap. Publication trends, collaboration networks, keyword co-occurrence patterns, and citation structures were examined to track how each field has evolved and to identify areas where their themes converge. We then used these convergent bibliometric signals to develop a focused mechanistic synthesis examining how EndMT and VM may interact within the broader framework of tumor vascular plasticity.

To our knowledge, this is the first comparative bibliometric analysis to examine EndMT and VM side by side within tumor biology. Using literature retrieved from Web of Science and Scopus, we sought to characterize the development, thematic structure, and research priorities of these two historically separate fields. We further examined their limited formal overlap and used convergent bibliometric signals to formulate a mechanistic framework for experimental investigation. Importantly, this framework is intended to generate testable hypotheses rather than to establish causal relationships that cannot be inferred from bibliometric data alone.

## 2 Bibliometric analysis

### 2.1 Data sources and search strategy

The flowchart illustrating the literature identification and screening process for WoSCC and Scopus is presented in **Figure 1**. To minimize discrepancies related to database updates, all searches were conducted on a single date: February 8, 2026. The analysis was restricted to publications from 2005 to 2025 written in English; only original research articles and reviews were included. Time-frame and document-type filtering were applied using the built-in search functions of WoSCC and Scopus during the initial retrieval phase. Records were exported in plain text format as full records with cited references for downstream processing.

**Figure 1:**
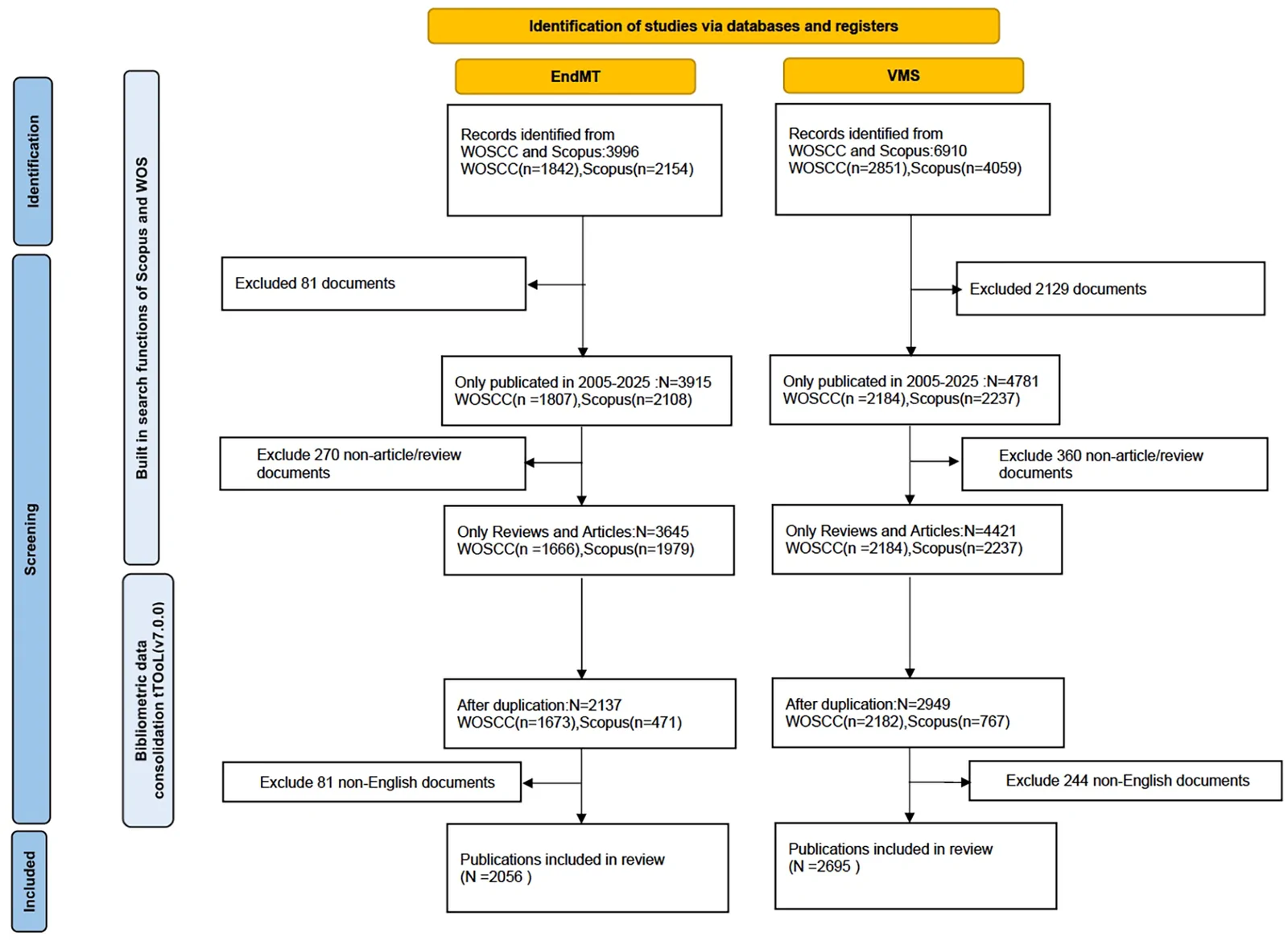
Flowchart of the literature identification and screening process for the EndMT and VM corpora.

### 2.2 Data processing and deduplication

Files exported from WoSCC and Scopus were processed using a custom-developed, open-source data fusion pipeline (available at: [https://github.com/elcdiehl-sketch/Bibliometric-Data-Consolidation-Tool-Enhanced-edition]), which builds upon and extends the foundational framework established by LeoMengTCM (Bibliometric Data Consolidation Tool, GitHub). The pipeline performs automated cross-database deduplication, normalizes field formats to ensure compatibility with downstream bibliometric software, screens for retracted articles, and supports advanced search filtering. Key improvements over prior implementations include more complete deduplication logic, standardized export formatting for compatibility with R/Biblioshiny, and integrated retraction screening. Following automated processing, the dataset was manually reviewed to confirm deduplication completeness. The final deduplicated corpora comprised 2,056 publications for the EndMT theme and 2,695 publications for the VM theme. Following database integration and deduplication, the EndMT and VM corpora were cross-screened using the predefined search terms and bibliographic metadata to identify publications addressing both topics. Candidate records were subsequently reviewed to confirm that EndMT and VM were explicitly discussed within a tumor-related context. This process identified 5 publications that satisfied the cross-theme inclusion criteria. These records were retained as an intersection corpus and subjected to independent qualitative analysis.

### 2.3 Inclusion and exclusion criteria

Publications were included if they were original research articles or review papers indexed in WoSCC or Scopus, published between 2005 and 2025, and written in English. Publications were excluded if they were conference abstracts, editorials, letters, or errata without substantive original content, or if they were identified as cross-database duplicates.

### 2.4 Bibliometric analysis

Bibliometric analyses were conducted in R (version 4.5.2) using the bibliometrix package and its Biblioshiny web interface (30). These tools were used to examine publication trends, authorship patterns, institutional and national collaboration networks, journal distribution, and citation structures. Keyword co-occurrence analysis and science mapping were performed using VOSviewer (31) (version 1.6.20), enabling visualization of thematic clustering and the evolution of research hotspots across the EndMT and VM corpora independently and within the intersection corpus. CiteSpace (version 6.4.1) was additionally employed for burst detection analysis to identify emerging research themes and track the temporal dynamics of influential publications within each corpus.

## 3 Results

### 3.1 Overview of the dual-theme research landscape

After the systematic search, we removed duplicates, screened for retractions, filtered by document type, and applied the language criteria. This left us with an EndMT corpus of 2,056 publications spanning 2005 to 2025. Using the same process, the VM corpus resulted in 2,695 publications across the same years. Together, these formed an analytical dataset of 4,746 unique records. When we compared the two corpora, we found 5 publications that addressed both EndMT and VM in an oncological setting. These papers formed a small intersection set, which we examined separately through qualitative analysis. A summary of the overall bibliometric characteristics of both corpora is provided in **Table 1**.

**Table 1:** Overview of the EndMT and VM bibliometric corpora.

Indicator	EndMT corpus	VM corpus
Total publications	2,056	2,695
Study period	2005–2025	2005–2025
Average citations/paper	42.46	38.06
Leading country	China (857, 41.7%)	China (1,137, 42.2%)
Top institution	Harvard Medical School (77)	Tianjin Medical University (131)
Top journal	Int J Mol Sci (59)	Oncotarget (62)
Intersection corpus	5 publications	

### 3.2 Annual scientific production and citation trends

#### 3.2.1 EndMT corpus

Annual publication output in the EndMT corpus shows a clear and steady rise across the study period (**Figure 2A**). The field began with only two papers in 2005 and reached 299 publications by 2025, a nearly 150-fold increase over two decades. Growth during 2005–2012 was modest but consistent, with annual output ranging from 2 to 37 papers. Activity increased substantially after 2015, reaching 73 publications, and accelerated further from 2019 onward. Annual counts from 2019 to 2025 were 146, 176, 217, 172, 200, 235, and 299, respectively. Although output dipped from 217 in 2021 to 172 in 2022, this appears to represent a short-term fluctuation rather than sustained deceleration. The exponential trendline further supports the overall upward trajectory, with 299 publications in 2025 marking the highest output during the study period.

**Figure 2:**
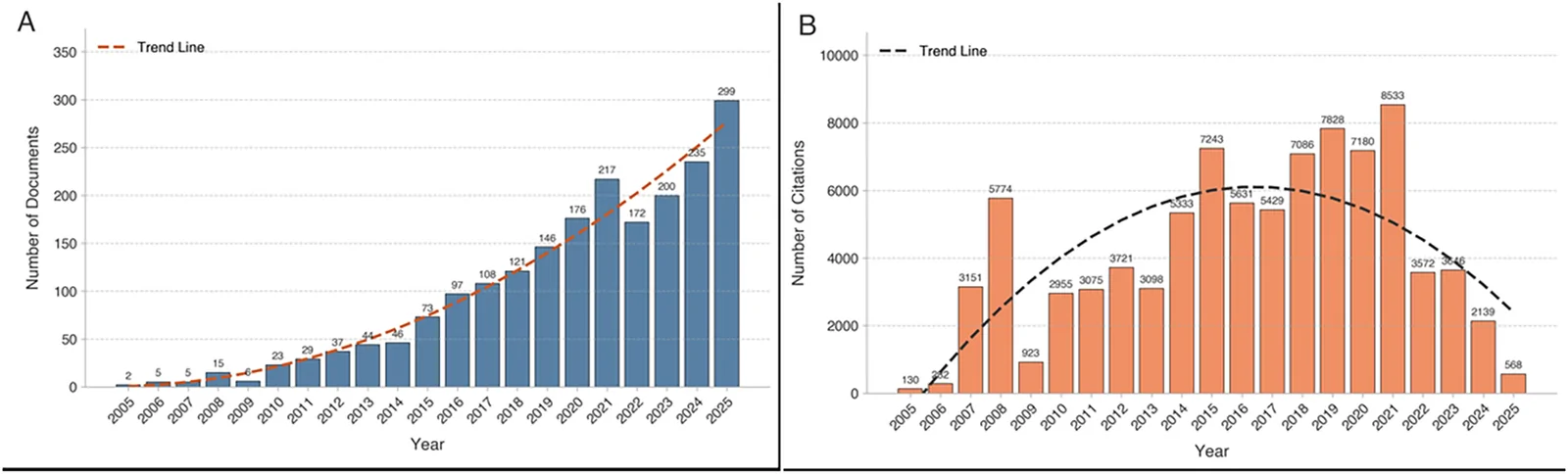
Annual scientific production **(A)** and citation trends **(B)** for the EndMT corpus (2005–2025). Bar heights represent annual publication counts; dashed line represents exponential trend. Citation data derived from Web of Science Core Collection and Scopus.

Citation patterns followed a different trajectory (**Figure 2B**). Annual citations increased from 130 in 2005 to a peak of 8,533 in 2021, with an overall upward trend despite some fluctuations. Citation counts subsequently declined to 3,572 in 2022, 3,646 in 2023, 2,139 in 2024, and 568 in 2025. This recent decrease likely reflects the well-known recency effect, as newer publications have had limited time to accrue citations rather than reduced scientific relevance. Thus, the divergence between the rising publication output (Panel A) and declining recent citation counts (Panel B) is consistent with a rapidly growing research field whose newest literature has not yet reached citation maturity.

#### 3.2.2 VM corpus

The VM corpus displayed a markedly different publication pattern from the EndMT corpus, reflecting VM’s longer and more established research history (**Figure 3A**). In 2005, the field already produced 46 papers, substantially more than the nascent EndMT output in the same year. Annual publications increased to 76–78 papers in 2007–2008, dipped slightly to 73–74 in 2009–2010, and rose to 94 in 2011 before falling to 79 in 2012. A clearer and more sustained expansion emerged from 2014 onward, with annual output increasing from 115 to a local peak of 170 in 2017. Publication counts then declined to 165 in both 2018 and 2019, increased to 189 in 2020, and fluctuated during 2021–2023 (176, 203, and 172, respectively). Output subsequently recovered to 195 in 2024 and reached a new high of 212 in 2025. Overall, unlike the smooth exponential growth of EndMT, VM represents a mature field with high but periodically fluctuating productivity.

**Figure 3:**
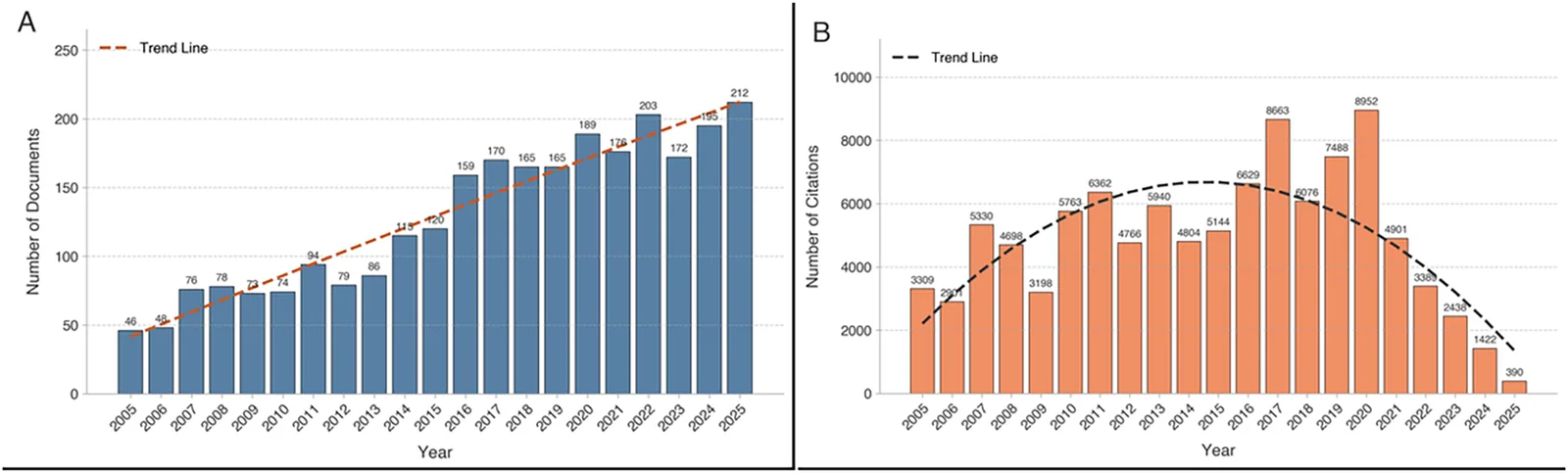
Annual scientific production **(A)** and citation trends **(B)** for the VM corpus (2005–2025).

Citation patterns in the VM corpus showed a similarly complex trajectory (**Figure 3B**). Annual citations began at 3,309 in 2005, declined to 2,901 in 2006, and rose sharply to 5,330 in 2007 and 4,698 in 2008. Citations then fluctuated between 4,766 and 6,629 during 2012–2016, with a peak of 8,663 in 2017. After declining to 6,076 in 2018, citations rose again to 7,488 in 2019 and reached the overall maximum of 8,952 in 2020. From 2021 onward, annual citations decreased progressively to 4,901, 3,389, 2,438, 1,422, and 390, respectively. As with the EndMT corpus, this recent decline primarily reflects the recency effect, as newer publications have had less time to accumulate citations, rather than a reduction in the field’s scientific influence.

### 3.3 Geographic distribution and international collaboration

#### 3.3.1 EndMT corpus

Analysis of national research output in the EndMT corpus identified 63 contributing countries. A small group of major research economies accounted for most of the work. China led by a wide margin with 857 publications (41.7% of the corpus). The United States followed with 421 publications (20.5%). Japan (105, 5.1%), Italy (87, 4.2%), and Germany (69, 3.4%) made up the next tier. The Netherlands (65, 3.2%), Canada and South Korea (47 each, 2.3%), France (41, 2.0%), and the United Kingdom (37, 1.8%) rounded out the top ten. Altogether, the first five countries produced roughly three-quarters of all EndMT publications.

The country collaboration map (**Figure 4A**) shows a network that leans heavily on a few central players. China, the United States, and Australia sit at the core. Many of the strongest links extend from China—to the United States, Germany, the Netherlands, Italy, and several partners across the Pacific. The density of these China-centered connections suggests that China is not only the largest contributor but also the main coordinating hub in global EndMT research.

**Figure 4:**
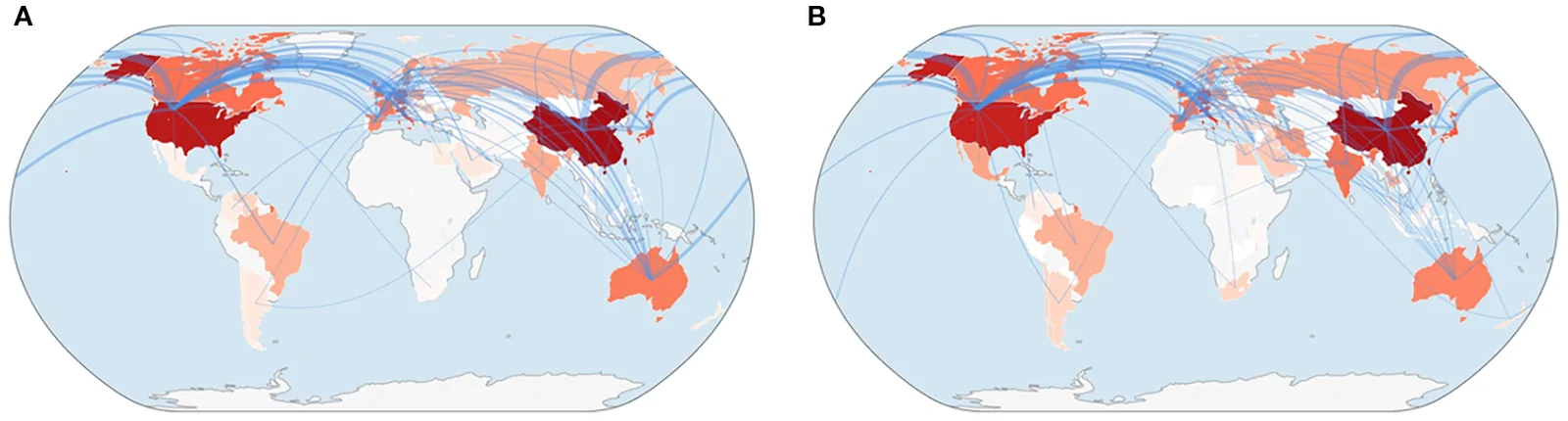
Country collaboration maps for the EndMT corpus **(A)** and VM corpus **(B)**. Node color intensity reflects publication volume; arc thickness reflects collaboration frequency.

#### 3.3.2 VM corpus

The VM corpus showed a similar pattern of geographic concentration. China again ranked first, with 1,137 publications (42.2%). The United States followed with 560 publications (20.8%). Italy contributed 196 papers (7.3%), Germany 163 (6.0%), and Japan 120 (4.5%). The rest of the top ten included the United Kingdom (90, 3.3%), Canada (79, 2.9%), India (76, 2.8%), Spain (73, 2.7%), and France (70, 2.6%). Compared with EndMT, the VM literature involved a wider set of countries. Italy, India, and Spain had a stronger presence, which fits the field’s longer history within both European and South Asian oncology communities.

The collaboration map for VM (**Figure 4B**) shows a structure that resembles that of EndMT, with China and the United States acting as the central hubs. One difference is the denser network across Europe. Italy, Germany, France, and the United Kingdom form a noticeable cluster, reflecting the role of European cancer research centers in advancing VM studies. Australia again appeared as a major trans-Pacific partner, a position it occupied in both datasets.

### 3.4 Institutional productivity and collaboration networks

#### 3.4.1 EndMT corpus

The institution collaboration network (**Figure 5A**) highlights Fudan University as the central hub, evident from its large node located in the lower-right of the map. This reflects its leading role in EndMT research. Other significant nodes include Harvard Medical School on the left, Sun Yat-sen University in the central red cluster, Wenzhou Medical University in the upper-left green cluster, and Guangzhou Medical University in the center-left orange cluster. Each of these institutions shows strong collaborative influence. Looking at the cluster distribution, mainland Chinese institutions make up the largest part of the network. They span several color-coded communities, collectively forming the backbone of the map. North American institutions, especially Harvard Medical School and Yale University, are grouped on the left, forming a distinct, high-impact cluster. European institutions serve as important connectors. Leiden University, positioned on the right in light blue, links the North American and Asian clusters. The German Centre for Cardiovascular Research (DZHK) sits in the lower-central region, bridging Chinese and European subcommunities. This positioning makes sense given EndMT’s strong ties to cardiovascular biology. Overall, the network shows a three-part structure: Chinese institutions dominate in volume, Harvard Medical School anchors the Western cluster, and European nodes act as key bridges across regions.

**Figure 5:**
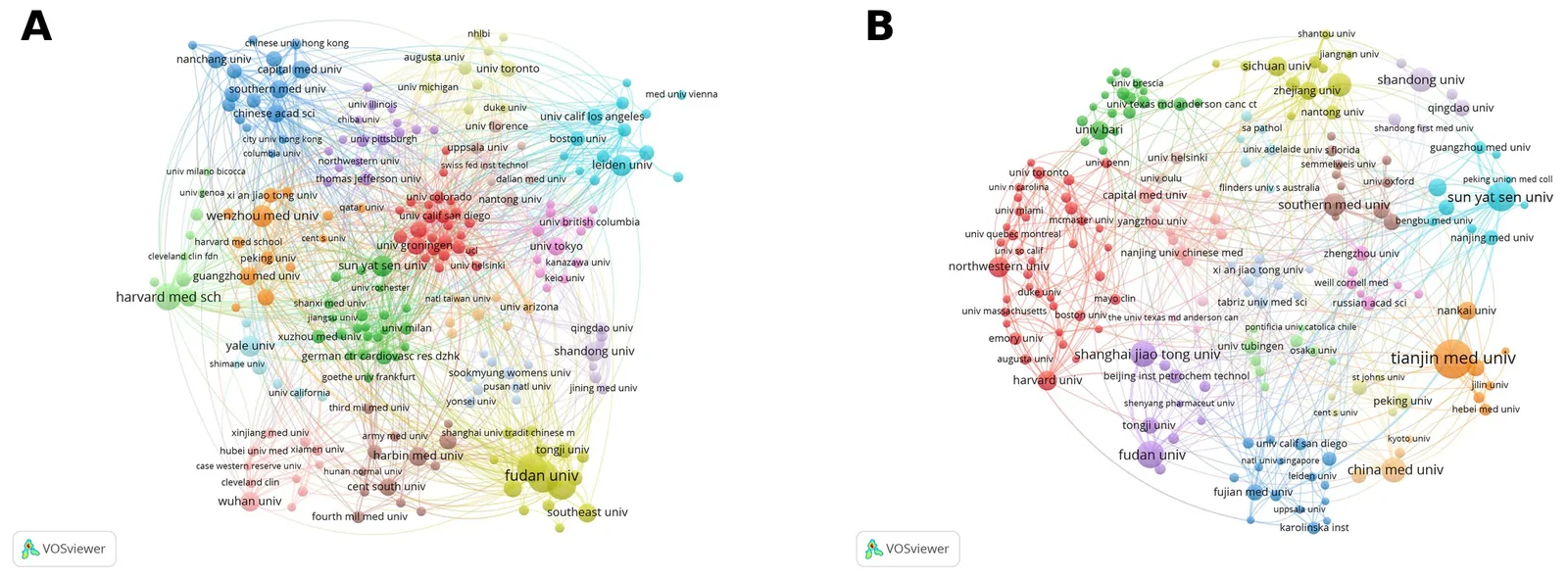
Institution collaboration networks for the EndMT corpus **(A)** and VM corpus **(B)**. Node size reflects total publication count; edge weight reflects co-authorship frequency; color denotes cluster community. Generated using VOSviewer.

#### 3.4.2 VM corpus

Institutional patterns in the VM corpus show a different form of concentration, with several major Chinese oncology centers taking the lead. Tianjin Medical University stands out the most, contributing 131 publications—nearly twice as many as the next institution. It is followed by Sun Yat-sen University (69), Fudan University (60), Shanghai Jiao Tong University (53), and Harvard University (49). The collaboration network (**Figure 5B**) mirrors this distribution. Tianjin Medical University and Sun Yat-sen University form the largest nodes, while China Medical University, Shandong University, and Sichuan University create an additional tier of highly active Chinese institutions. A separate North American cluster—anchored by Northwestern University, Harvard University, and Duke University—appears on the left side of the network, connected to the Chinese institutions through shared collaborations. Tianjin Medical University’s placement reflects its sustained focus on VM in tumor pathology and its work on prognostic markers, especially in lung and hepatocellular carcinoma. The citation burst analysis (**Figure 6B**) supports this view. Tianjin Medical University shows the strongest institutional burst (strength 18.45, 2012–2018), highlighting its central role during the mid-phase of VM research.

**Figure 6:**
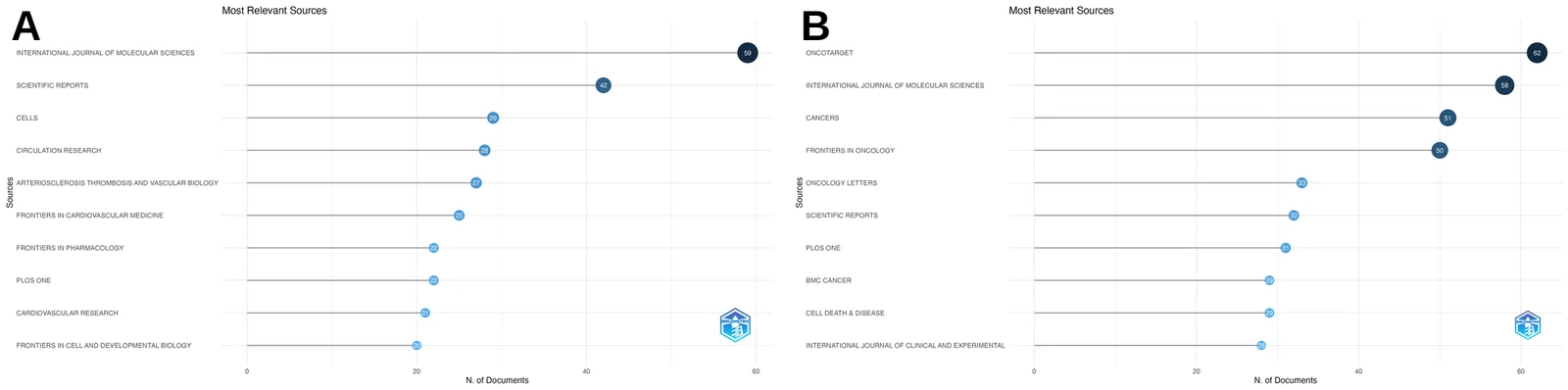
Most relevant publication sources in the EndMT corpus **(A)** and VM corpus **(B)**. Bar length indicates number of publications; bubble size proportional to publication count.

### 3.5 Most relevant publication sources

#### 3.5.1 EndMT corpus

The ten most productive journals in the EndMT corpus are shown in **Figure 6A**. International Journal of Molecular Sciences published the largest number of papers (59), followed by Scientific Reports (42) and Cells (29). Several high-impact cardiovascular journals appear prominently as well. Circulation Research (28), Arteriosclerosis, Thrombosis, and Vascular Biology (27), Frontiers in Cardiovascular Medicine (25), and Cardiovascular Research (21) highlight the field’s long-standing ties to vascular and cardiac biology. It is also notable that broad-scope, open-access journals sit alongside these specialty titles. Their presence suggests that EndMT work is being shared across both discipline-focused and cross-disciplinary outlets. This pattern fits the way the field has expanded into oncology, fibrosis, and immunology in recent years.

#### 3.5.2 VM corpus

The VM corpus showed a very different journal landscape, one that is shaped largely by oncology-focused open-access outlets (**Figure 6B**). Oncotarget was the most common venue with 62 publications. It was followed closely by the International Journal of Molecular Sciences (58), Cancers (51), and Frontiers in Oncology (50). All four are broad oncological journals with open-access models. The rest of the top ten included Oncology Letters (33), Scientific Reports (32), PLOS ONE (31), BMC Cancer (29), Cell Death & Disease (29), and the International Journal of Clinical and Experimental Pathology (28).This pattern stands in sharp contrast to what we observed for EndMT. Cardiovascular specialty journals are almost entirely absent here, while pan-cancer open-access platforms dominate the list. The distribution makes VM’s identity clear: it is rooted firmly in oncology and covers a wider range of cancer types than EndMT, which grew out of a more cardiovascular-centered research tradition.

### 3.6 Most globally cited documents

#### 3.6.1 EndMT corpus

The ten most widely cited publications in the EndMT corpus are shown in **Figure 7A**. At the top of the list is Wynn’s 2008 review in Journal of Pathology, cited 3,454 times. It remains a key fibrosis paper and has shaped much of the mechanistic thinking behind EndMT in tissue remodeling. The next most cited work is the 2007 Nature Medicine study by Zeisberg, Tarnavski, and colleagues, with 1,862 citations. This is the seminal EndMT paper in cardiac fibrosis. A 2014 Science Signaling review by Gonzalez and Medici follows with 1,437 citations and provides one of the field’s broadest overviews of EndMT signaling. Another highly cited contribution is the 2007 Cancer Research article by Zeisberg, Potenta, Xie, Zeisberg, and Kalluri. With 826 citations, it ranks fifth and stands as the defining lineage-tracing study for cancer-associated fibroblasts, firmly placing it among the core references in oncological EndMT research. The Three-Field Plot (**Figure 8A**) adds another layer to this picture. The most frequently cited references cluster around themes such as fibrosis, endothelial–mesenchymal transition, angiogenesis, and endothelial cells. These links show how foundational review papers continue to anchor the vocabulary and conceptual structure of the field.

**Figure 7:**
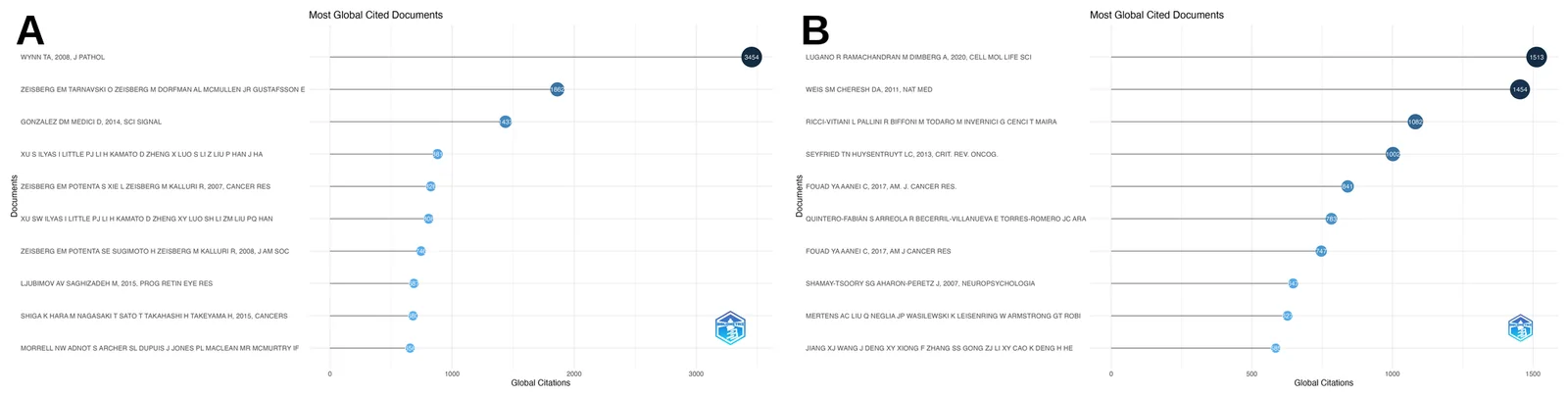
Most globally cited documents in the EndMT corpus **(A)** and VM corpus **(B)**. Bubble size represents global citation count.

**Figure 8:**
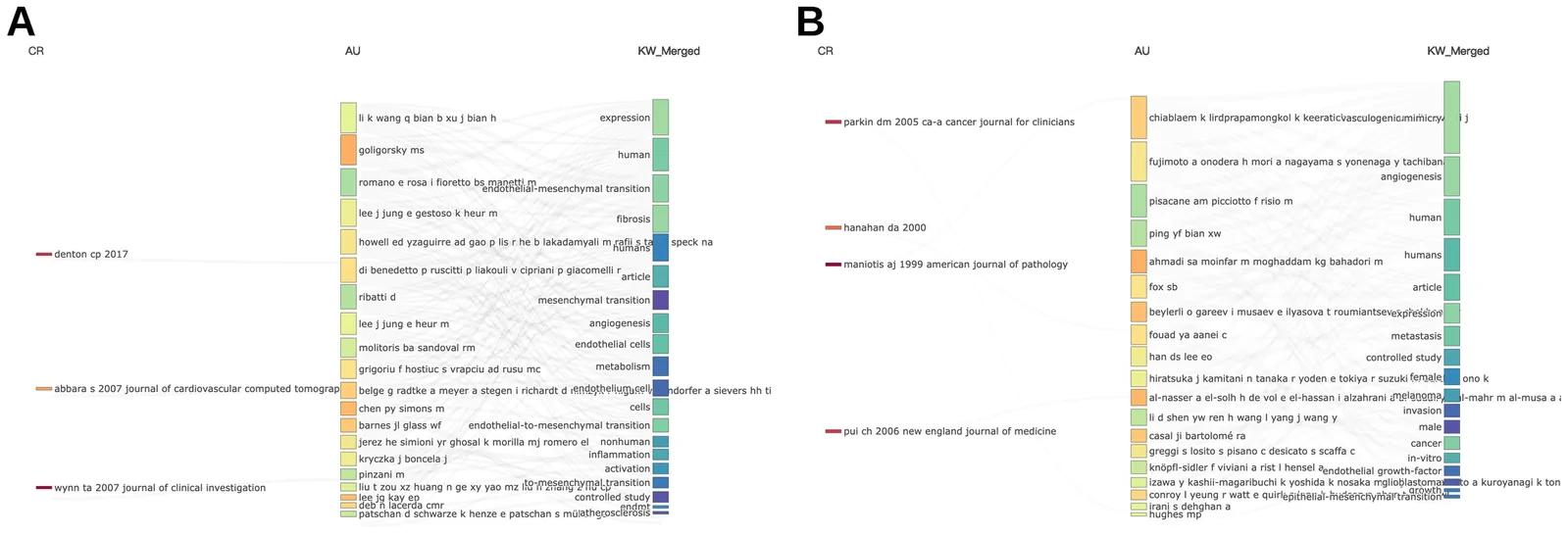
Three-field plots for the EndMT corpus **(A)** and VM corpus **(B)**. Left axis: most cited references; center axis: most productive authors; right axis: dominant merged keywords. Line thickness reflects co-occurrence or co-citation strength.

#### 3.6.2 VM corpus

The VM corpus showed a different citation landscape, shaped by its own early literature (**Figure 7B**). The most cited paper was the review by Lugano, Ramachandran, and Dimberg (2020, Cellular and Molecular Life Sciences), which had 1,513 citations. It offered a broad overview of tumor angiogenesis and placed VM within the wider framework of tumor vascularization. Close behind was the article by Weis and Cheresh (2011, Nature Medicine) with 1,454 citations, a key piece on angiogenesis and vascular normalization. Ricci-Vitiani, Pallini, Biffoni and colleagues ranked third with 1,082 citations, followed by the review from Seyfried and Huysentruyt (2013, Critical Reviews in Oncology/Hematology) with 1,002 citations. Fouad and Aanei (2017, American Journal of Cancer Research) appeared twice in the top ten because of slight differences in how the paper was indexed across datasets—yielding tallies of 841 and 747 citations. Both entries point to the heavy and repeated citation of this review on EMT hallmarks. The Three-Field Plot (**Figure 8B**) highlighted the paper by Maniotis et al. (1999, American Journal of Pathology) as a central reference node in the VM network, consistent with its status as the original VM discovery paper. Other influential nodes included Parkin (2005), Hanahan and Weinberg (2000), and Pui (2006), reflecting how the VM literature initially intersected with broader work on cancer epidemiology, hallmarks, and early studies in hematologic malignancies.

### 3.7 Keyword co-occurrence network and thematic clustering

#### 3.7.1 EndMT corpus

Keyword co-occurrence analysis of the EndMT corpus (**Figure 9A**) revealed three distinct thematic clusters. The largest and most tightly connected cluster is shown in red. It gathers the core molecular and pathological terms associated with EndMT, including endothelial-to-mesenchymal transition, fibrosis, TGF-β, growth factor beta, oxidative stress, microRNA, Snail, collagen, apoptosis, migration, macrophages, and induction. Together, these terms capture the central focus of EndMT research, sitting at the crossroads of vascular biology, molecular signaling, and tissue injury responses. The green cluster appears on the left side of the map. It reflects the experimental and marker-based aspects of EndMT studies. Terms in this cluster include vascular endothelial cadherin, alpha-smooth muscle actin, vimentin, pathology, immunohistochemistry, signal transduction, epithelial-mesenchymal transition, cell migration, and western blotting. This grouping highlights the experimental tools and mesenchymal marker framework that researchers rely on to identify and validate EndMT in the lab. On the right side of the map, the blue cluster focuses on transforming growth factor beta and myofibroblasts. Associated terms include cell transformation, endothelium cell, and cardiovascular disease. This cluster represents the link between endothelial activation and pathological tissue remodeling, emphasizing the fibrotic and cardiovascular dimensions of EndMT. Notably, the green cluster sits between the red and blue clusters. Carrying both vascular marker terms and experimental methodology terms, it forms a region of the keyword landscape that overlaps most closely with vascular mimicry (VM) biology.

**Figure 9:**
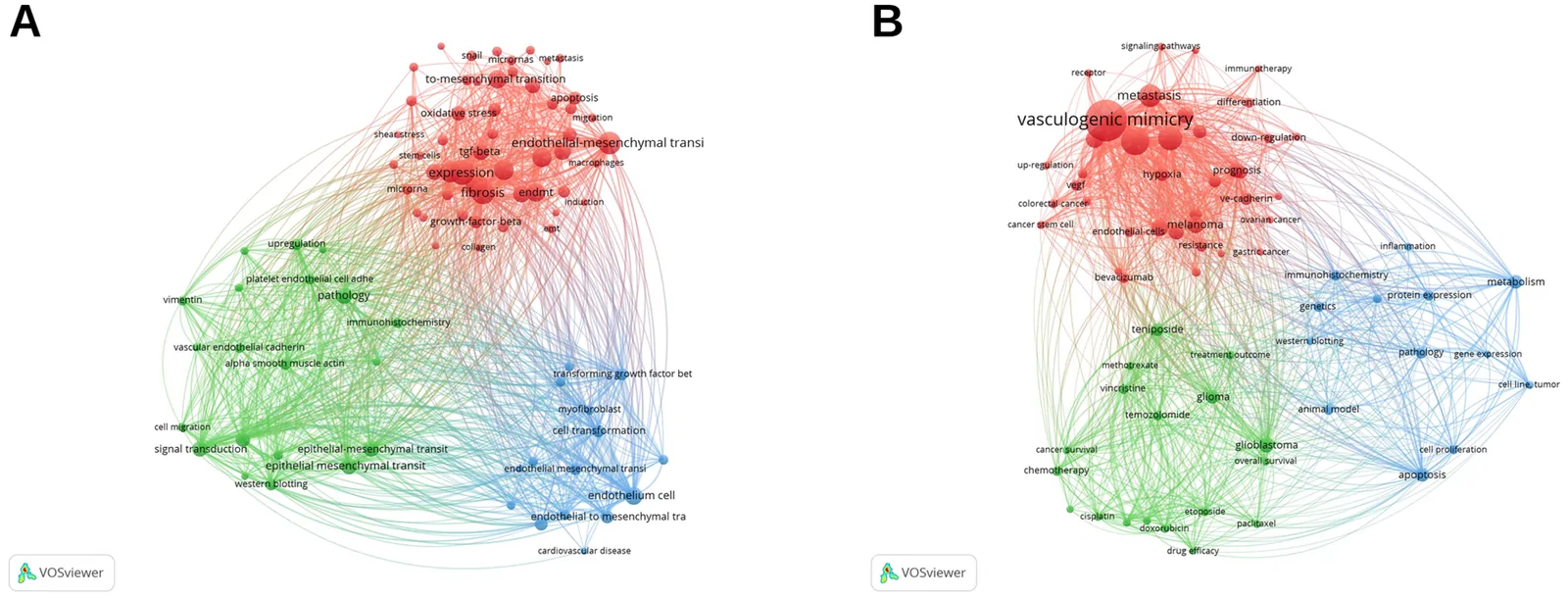
Keyword co-occurrence networks for the EndMT corpus **(A)** and VM corpus **(B)**, generated using VOSviewer. Node size reflects keyword frequency; edge weight reflects co-occurrence strength; color denotes thematic cluster.

#### 3.7.2 VM corpus

The keyword co-occurrence analysis of the VM corpus (**Figure 9B**) shows three well-defined thematic clusters, each with its own functional focus. The red cluster is the dominant group, centered on vasculogenic mimicry and metastasis. Around these core nodes are hypoxia, melanoma, prognosis, endothelial cells, bevacizumab, resistance, VE-cadherin, cancer stem cells, signaling pathway, immunotherapy, differentiation, down-regulation, and several tumor types including colorectal, ovarian, and gastric cancer. This cluster captures how deeply VM is embedded in tumor biology and how closely it aligns with work on anti-angiogenic therapies across a broad range of malignancies. The green cluster, located in the lower left, gathers the chemotherapy and drug-resistance dimension of VM research. It brings together teniposide, vincristine, methotrexate, temozolomide, cisplatin, etoposide, doxorubicin, paclitaxel, glioma, glioblastoma, overall survival, and treatment outcome. These terms reflect the established overlap between VM and chemotherapy resistance, particularly in neuro-oncology and hematological malignancies. The blue cluster occupies the right portion of the map and is anchored by experimental and mechanistic vocabulary: metabolism, inflammation, protein expression, immunohistochemistry, pathology, gene expression, western blotting, apoptosis, and cell proliferation. This cluster represents the experimental infrastructure underlying mechanistic VM studies and provides the methodological counterpart to the clinical vocabulary of the red cluster.

One feature in the red cluster stands out. VE-cadherin sits next to endothelial cells and resistance, creating a link between the core VM oncology terms and the vocabulary of endothelial biology. Its downregulation is a well-known sign of endothelial activation in EndMT, and its appearance here alongside resistance and stemness suggests that VM research is beginning to fold endothelial plasticity programs into its molecular thinking. This overlap marks a conceptual point of contact between the two areas and sets the stage for the mechanistic discussion in Section 4.

### 3.8 Keyword and citation burst detection: research evolution

#### 3.8.1 EndMT corpus

Citation burst analysis using CiteSpace highlighted the top 25 keywords with the strongest burst intensity over the study period (**Figure 10A**). This analysis reveals how research priorities in EndMT evolved through three distinct phases. The first phase, spanning roughly 2005–2014, laid the biological groundwork for the field. Early burst terms such as endothelium (strength 6.70, 2005–2016) and epithelial–mesenchymal transition (6.58, 2006–2013) reflected EndMT’s roots in vascular biology and its conceptual borrowing from studies on epithelial plasticity. Soon after, transformation (6.89, 2008–2015) and differentiation (8.67, 2009–2015) emerged, capturing the consolidation of vocabulary around cellular identity changes. Fibrosis also appeared during this period (5.26, 2008–2014), signaling how quickly pathological tissue remodeling became a central concern. The strongest burst in the dataset belongs to fibroblasts (18.72, 2010–2017). This term rose alongside progenitor cells (12.38, 2010–2017), *in vitro* (11.14, 2010–2018), *in vivo* (8.21, 2010–2019), cardiac fibrosis (7.94, 2010–2018), and growth factor (6.69, 2010–2014). Their simultaneous appearance marks a phase when EndMT was increasingly recognized as a source of pathological fibroblast populations. Growth factor signaling and validation across both *in vitro* and *in vivo* systems were key drivers of this understanding. Cell transformation (6.60, 2011–2016) and heart (6.37, 2012–2017) extended this mechanistic focus into organ-specific contexts. The second phase, roughly 2013–2020, shifted attention toward disease-specific settings and signaling pathways. Renal fibrosis (11.56, 2014–2017) and molecular mechanisms (8.74, 2014–2018) indicated that the field was expanding beyond cardiac applications. TGF-β (17.87, 2015–2017), the second strongest burst overall, reinforced its role as a central mechanistic driver. Terms such as cytology (5.64, 2016–2020) and signaling pathway (5.57, 2016–2021), with pathway (5.42, 2017–2020), reflect the growing methodological and conceptual depth in studying EndMT. The most recent burst phase (2020–2025) focuses on mechanosensing, vascular biology, and immune mediators. Shear stress (5.27, 2020–2023) and flow (5.65, 2021–2022) show a rising interest in hemodynamic regulation. Vascular calcification (5.41, 2021–2023) highlights expanding disease applications, while tumor necrosis factor (5.95, 2022–2025) and differential gene expression (6.69, 2023–2025) point to a shift toward inflammatory drivers and transcriptomic approaches. Taken together, these bursts trace a clear path of maturation in EndMT research. The field began with defining core cellular and molecular mechanisms, expanded into disease-specific contexts, and is now integrating mechanosensory, immune–inflammatory, and transcriptome-level investigations.

**Figure 10:**
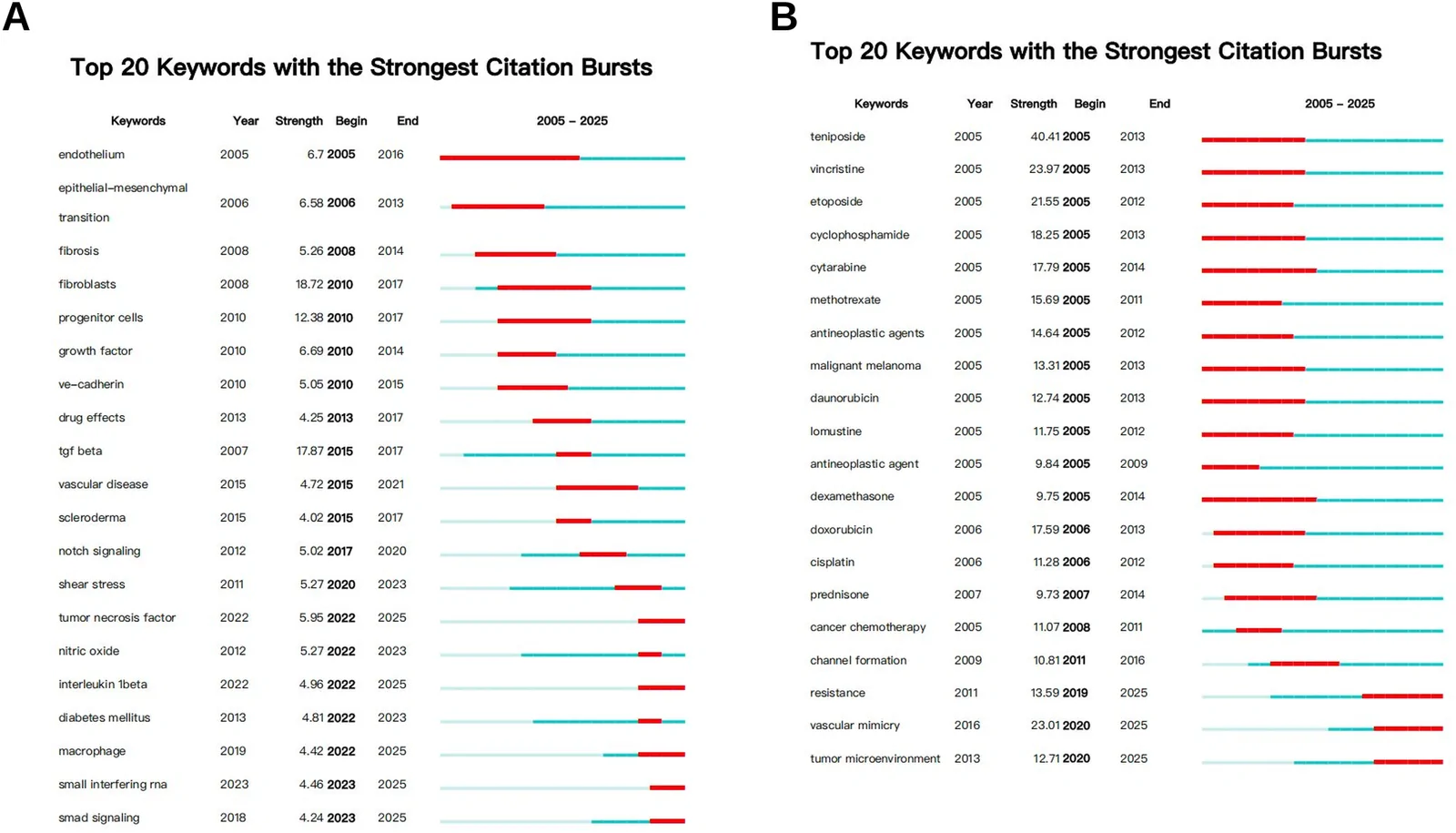
Citation burst detection: EndMT corpus **(A)** and VM corpus **(B)**, generated using CiteSpace. Red bars indicate burst periods; teal lines indicate the full study period. Burst strength values are shown for each keyword.

#### 3.8.2 VM corpus

Citation burst analysis of the VM corpus (**Figure 10B**) shows a temporal pattern that reflects the field’s earlier start and its distinct research trajectory. The evolution falls into three phases. The first phase (burst begin 2005–2008) is the most crowded and is dominated by chemotherapy-related terms. Teniposide shows the strongest burst in the entire dataset (40.41, 2005–2013), followed by vincristine (23.97), etoposide (21.55), cyclophosphamide (18.25), cytarabine (17.79), and doxorubicin (17.59, 2006–2013). Other terms include methotrexate (15.69), antineoplastic agents (14.64), malignant melanoma (13.31), daunorubicin (12.74), cancer chemotherapy (11.07), lomustine (11.75), cisplatin (11.28, 2006–2012), antineoplastic agent (9.84), dexamethasone (9.75), and prednisone (9.73, 2007–2014). The sheer density of this cluster stands out. It points back to the field’s roots in hematologic oncology and melanoma, where combination chemotherapy was the dominant setting, and where VM first drew attention as a mechanism of resistance in drug-treated tumor cells. A shorter transitional phase follows, centered on a single but important term: channel formation (10.81, 2011–2016). This marks a shift in focus. Rather than staying at the clinical level, researchers began to look more closely at the structure and function of VM channels themselves. It is a clear move toward mechanism. The most recent phase (2019–2025) shows where the field is now heading. Vascular mimicry has the second strongest burst overall (23.01, 2020–2025), pointing to renewed and more focused attention on the phenomenon itself. Alongside it are resistance (13.59, 2019–2025) and tumor microenvironment (12.71, 2020–2025). Together, these terms define the current research front. Their co-occurrence suggests that VM is now being framed through microenvironmental influences and therapy resistance, bringing the field into the same conceptual space where EndMT is increasingly being studied at the mechanistic level.

### 3.9 Thematic map and research frontier analysis

In the EndMT thematic map (**Figure 11A**), three patterns stand out. The first is a clear motor-theme cluster in the upper-right quadrant, organized around TGF-β signaling, fibrosis, and vascular endothelial biology. The cluster is dense, which means these topics are frequently cross-referenced within the EndMT literature. Its high centrality shows that this group of themes acts as a structural backbone for the field. A second cluster appears in the high-centrality but low-density quadrant. This area brings together concepts related to the tumor microenvironment, cancer-associated fibroblasts, and stromal remodeling. These topics interact with many parts of the map but have not yet formed fully independent research programs. Their centrality is what makes them interesting: they lie at the meeting point between EndMT and oncology, and they connect long-established mechanistic work with newer translational directions. The third cluster sits in the lower-left quadrant and reflects early work on immunological aspects of EndMT and endothelial metabolic reprogramming. These themes have not yet accumulated enough internal citations to form cohesive clusters, but their presence hints at where growth in the field may occur next.

**Figure 11:**
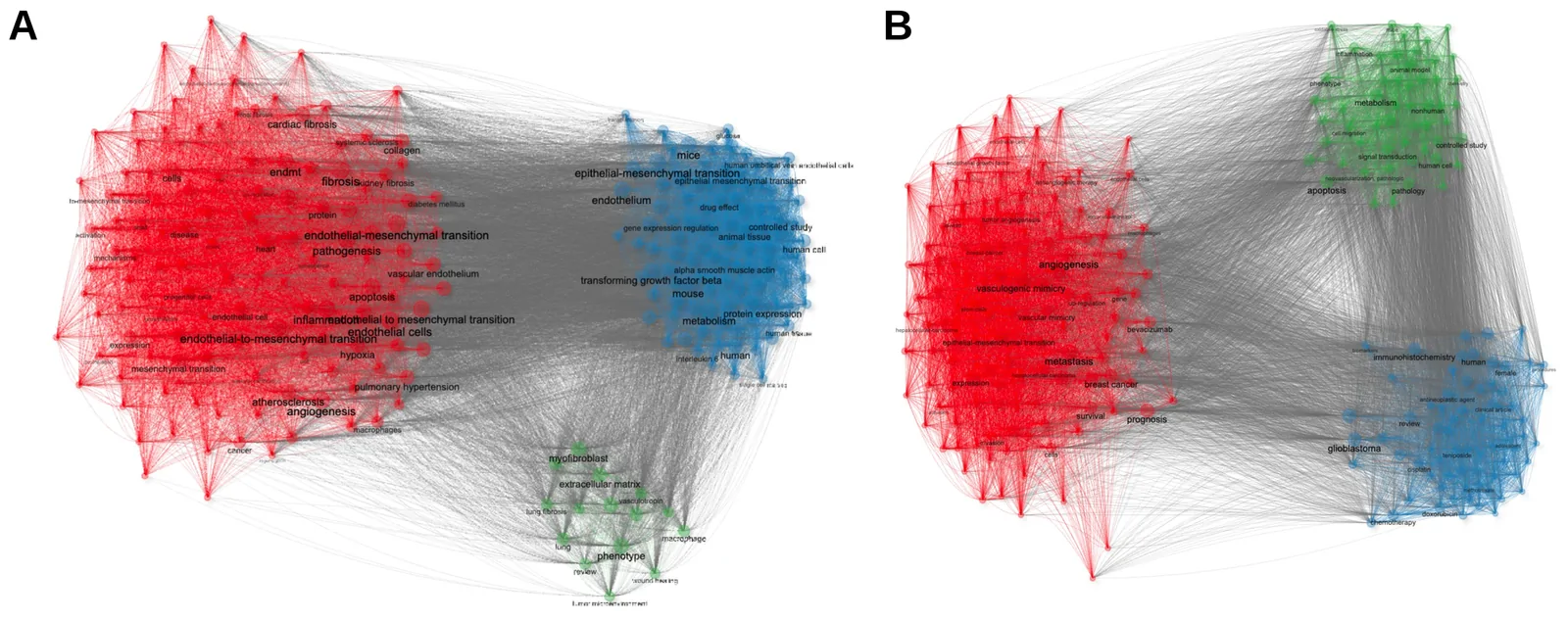
Thematic maps for the EndMT corpus **(A)** and VM corpus **(B)**, generated using bibliometrix. Quadrants represent motor themes (upper right), niche themes (upper left), emerging/declining themes (lower left), and basic themes (lower right). Bubble size reflects theme relevance.

In the VM thematic map (**Figure 11B**), the motor-theme quadrant is anchored by a cluster built around epithelial–mesenchymal transition, hypoxia, and tumor angiogenesis. Its placement reflects how far mechanistic VM research has matured and how seamlessly it now sits within the wider frameworks of cancer biology. A smaller, high-density but low-centrality cluster emerges alongside it. This niche group is composed mainly of glioblastoma-oriented VM studies and work on chemotherapy resistance in neuro-oncology—topics that form a coherent internal conversation yet remain only loosely tied to the broader VM corpus. What stands out most, however, is the basic-theme region. It gathers terms such as tumor microenvironment, immune infiltration, and stromal interactions, and in doing so reproduces the same underdeveloped perimeter that appears in the EndMT map. The fact that the two maps converge in precisely this region—both pointing toward the tumor microenvironment and stromal remodeling as structurally central but still weakly consolidated areas—provides the bibliometric justification for the mechanistic synthesis in Section 4.This overlap is what motivated the choice of the three mechanistic axes discussed there: shared TGF-β/HIF-1α/Notch signaling, the stromal-remodeling interface, and partial transition states. These are the points where the two fields are most likely to intersect mechanistically, and also the points where the existing literature is thinnest—making them scientifically the most promising places to push the integration forward.

### 3.10 Cross-theme intersection corpus analysis

Cross-theme review of the two final corpora initially identified 8 publications whose DOIs appeared in both the EndMT and VM retrieval sets. Each record was subsequently evaluated against a pre-specified double inclusion criterion: a publication was retained in the intersection corpus only if both EndMT and VM constituted central, independently developed arguments of the paper — not merely cited or incidentally mentioned phenomena. Applying this criterion reduced the intersection corpus to 5 publications that genuinely address both processes in an oncological setting. The remaining 3 records — two extracellular vesicle reviews and one PARP-1/melanoma study — were retained in their respective single-theme corpora, as EndMT and VM each appeared in a supporting rather than primary analytical role; their exclusion reflects stricter methodological standards rather than a retrieval failure.

This refined group of 5 papers both highlights the research gap noted in Section 1 and provides the empirical foundation for the comparative design used in this study. Five papers represent less than 0.105% of the full 4,746-publication dataset. The proportion is striking, given that EndMT and VM share several molecular drivers — TGF-β, HIF-1α, Notch, and VE-cadherin — yet the research communities studying them have worked with very little interaction.

A closer look at these 5 papers shows that they span original experimental research and integrative review, ranging from PDGF-driven EndMT conferring anti-VEGF resistance in glioblastoma (Nature Communications, 2018) to a 3D bioprinted neurovascular unit model revealing co-occurring EndMT cell populations and VM-like structures in GBM (Advanced Healthcare Materials, 2024). They cluster between 2013 and 2024, with four of the five published after 2018, indicating that recognition of the EndMT–VM intersection is only beginning to emerge. None of the papers used bibliometric tools, and none attempted to describe the broader research landscape of either field. This absence underscores the novelty of the dual-theme bibliometric approach taken here.

When considered alongside the burst detection results — where epithelial–mesenchymal transition (2018–2023) and the tumor microenvironment (2020–2025) appear as frontier VM keywords — the intersection analysis suggests that EndMT and VM are starting to converge in the literature. The idea is no longer purely conceptual; it is becoming visible in publication patterns. The mechanistic synthesis in Section 4 is designed to help reinforce this shift by outlining a shared molecular framework built around the established priorities of both research communities.

## 4 Mechanistic analysis: molecular convergence of EndMT and vasculogenic mimicry

The bibliometric findings reveal that EndMT and VM have evolved as parallel yet largely non-communicating research programs. At the same time, their keyword co-occurrence topologies (**Figure 9**), citation-burst trajectories (**Figure 10**), thematic-map placements (**Figure 11**), and the recent emergence of shared frontier terms — tumor microenvironment, epithelial–mesenchymal transition, resistance, and TGF-β—highlight areas of potential mechanistic convergence. The co-occurrence networks (**Figure 9**) show that both corpora contain peripheral clusters related to extracellular-matrix dynamics, stromal remodeling, and cellular plasticity. These shared themes provide a conceptual link between the two fields, although bibliometric similarity alone cannot establish a biological relationship. This signal is reinforced by the burst analysis (**Figure 10**), which shows that the most recent high-intensity burst terms in both datasets increasingly involve the tumor microenvironment, therapeutic resistance, and EMT-related concepts. This pattern suggests that the two research communities are moving toward related conceptual frontiers, although they have approached these questions from distinct disciplinary trajectories. The thematic maps (**Figure 11**) provide a complementary structural perspective. In both corpora, the basic-theme quadrants — regions of high centrality but low density, representing domains that are foundational yet insufficiently developed — overlap almost precisely in the area of stromal remodeling and microenvironmental signaling. Taken together, these bibliometric patterns informed the mechanistic synthesis presented below, which focuses on three candidate areas of interaction: overlapping upstream signaling pathways, the stromal-remodeling interface, and partial or hybrid cellular states.

### 4.1 Shared upstream signal architecture: TGF-β, HIF-1α, and Notch

One of the most prominent areas of potential mechanistic convergence between EndMT and VM involves TGF-β, hypoxia/HIF-1α, and Notch-related signaling. Several layers of bibliometric evidence point to these pathways as recurrent themes within the two literatures. In the EndMT co-occurrence network (**Figure 9A**), terms related to TGF-β, hypoxia, and Notch cluster within the densely connected blue and red regions, reflecting the long-standing dominance of these pathways in the EndMT literature. Burst detection adds another angle: in the EndMT corpus (**Figure 10A**), TGF-β shows a burst strength of 17.87 during the 2015–2017 consolidation period, representing the second strongest burst in the entire EndMT dataset. More recent bursts during 2020–2025 shifted toward mechanosensing, hemodynamic regulation, and immune-related terms such as tumor necrosis factor and differential gene expression, suggesting that EndMT research has increasingly expanded beyond its established signaling pathways toward broader cellular and microenvironmental contexts. The VM burst analysis (**Figure 10B**) shows a parallel trend. The most prominent recent bursts—vascular mimicry (23.01, 2020–2025), resistance (13.59, 2019–2025), and tumor microenvironment (12.71, 2020–2025)—indicate increasing attention to tumor-cell plasticity, treatment adaptation, and microenvironmental regulation. However, these bibliometric patterns should not be interpreted as evidence that these processes are driven by a single shared TGF-β/HIF-1α/Notch signaling hub. Rather, they identify partially overlapping areas of research interest that warrant examination at the mechanistic level. The thematic maps further support this interpretation. In the EndMT map (**Figure 11A**), TGF-β signaling occupies the motor-theme quadrant, indicating a mature and well-developed research area within the field. In the VM map (**Figure 11B**), EMT and hypoxia occupy a similarly prominent position, highlighting their central roles in the VM literature. These parallel patterns suggest that both fields have developed around related concepts of cellular plasticity and microenvironmental regulation, but the similarity of their bibliometric structures does not by itself establish a direct biological connection. Taken together, the bibliometric findings identify TGF-β, hypoxia/HIF-1α, and Notch-related signaling as candidate areas of mechanistic overlap between EndMT and VM rather than as a unified molecular backbone. Experimental evidence is therefore required to determine whether these pathways independently regulate endothelial and tumor-cell plasticity or whether their convergence within particular tumor microenvironments produces functional interactions between the two processes.

TGF-β signaling is one of the most familiar drivers of EndMT. Most studies focus on TGF-β1 and TGF-β2, but TGF-β3 acts through the same general routes. All three ligands can trigger the Smad2/3 pathway and several non-canonical branches, including MAPK, PI3K/Akt, and Rho GTPases (25, 26). Through these signals, endothelial traits fade. Markers such as VE-cadherin, CD31/PECAM-1, Tie1, Tie2, and vWF decline, and the cells begin to shift toward a mesenchymal profile (16). This shift is steered by transcription factors like Snail, Slug, Twist, ZEB1, SIP1/ZEB2, and LEF-1, which collectively reshape the endothelial program (26). 

Snail is a central driver of TGF-β2-induced EndMT; it is required for ACTA2 induction, though its sufficiency depends on co-occurring signals that prevent GSK-3β-mediated inactivation of Snail protein. ACTA2 in turn represses VE-cadherin (CDH5) expression. Slug also contributes, though its influence tends to follow Snail and reinforces the transition rather than initiating it (32). An additional layer of control comes from epigenetic regulation (33). EZH2-mediated H3K27 trimethylation silences endothelial-specific genes such as CDH5, while DNMT3A-mediated promoter hypermethylation suppresses KLF4 expression. Together, these epigenetic changes reinforce transcriptional reprogramming toward a mesenchymal state (33, 34). 

The same set of transcription factors — Snail, Twist, and ZEB1 — is also essential for VM in tumor cells (5, 35). When these factors are active, tumor cells begin to express VE-cadherin and EphA2, which confers the ability to form VM structures (36). Knocking down any of these regulators has been shown to impair VM formation in multiple tumor models.

The tumor microenvironment adds another layer to how TGF-β drives EndMT. Tumor-derived endothelial cells respond to TGF-β2 with a much stronger α-SMA induction than normal endothelial cells, and contain subpopulations with different degrees of EndMT flexibility — variation that does not appear in regular vascular endothelium (27). This heightened sensitivity reflects the shifted epigenetic and transcriptional state of the microenvironment and helps explain why EndMT tends to cluster at the tumor–stroma border rather than appearing uniformly in more distant vessels. TGF-β has also been implicated in VM through paracrine and microenvironmental mechanisms. These findings raise the possibility that TGF-β-related stromal signaling may contribute to the permissiveness of neighboring tumor cells for VM; however, direct evidence linking EndMT-associated TGF-β signaling to VM initiation remains limited (28). 

Hypoxia acts as a central driver of both pathways, largely through HIF-1α. In EndMT, HIF-1α can activate Snail directly by binding to hypoxia-response elements within the SNAI1 promoter. It also strengthens TGF-β/Smad signaling; loss of RHOJ prevents formation of the Twist–Snail repressor complex, leaving the pathway unobstructed. In addition, HIF-1α engages the Notch–LOX axis, which helps maintain the mesenchymal transcriptional state once it is established (37). Hypoxia-induced EndMT is accompanied by a familiar set of molecular changes: α-SMA rises, VE-cadherin falls, and the Wnt/β-catenin and NF-κB pathways activate to reinforce the transition (37, 38). In VM, HIF-1α stands out as one of the strongest drivers of the phenotype. It can bind several hypoxia-response elements upstream of the VE-cadherin promoter in tumor cells, driving ectopic expression. At the same time, HIF-1α elevates EphA2, Twist, Nodal, and COX-2, thereby promoting a molecular program associated with VM competence (35, 39). 

Hypoxia also raises levels of the anti-apoptotic protein Bcl-2, adding a further layer of support for VE-cadherin expression in melanoma cells through a secondary feed-forward loop that helps sustain the VM-related program once initiated (40). The HIF-1α–EphA2–Laminin-5γ2 pathway represents another key driver of VM: in this route, MMP-2 cleaves Laminin-5γ2 to produce the γ2′ and γ2x fragments, which promote cell migration — a cleavage step essential for VM channel formation across several solid tumor types (39). 

Notch signaling requires a particularly context-dependent interpretation. In physiological angiogenesis, Notch receptors and ligands—including Notch1, Notch4, DLL4, and Jagged1—play central roles in endothelial cell fate determination, tip–stalk organization, arterial specification, vascular maturation, and communication with neighboring cells (41–43). Importantly, different receptor–ligand combinations can produce distinct or even opposing endothelial responses. DLL4–Notch signaling is central to angiogenic sprouting and arterial endothelial specification, whereas Jagged1 can modulate or antagonize DLL4-dependent responses (42, 43). In pathological settings, altered Notch signaling has also been implicated in EndMT and tumor-cell plasticity (15, 26, 41). Thus, Notch should not be interpreted simply as an aberrantly activated EndMT pathway; its effects on endothelial or tumor-cell plasticity are likely to depend on receptor–ligand configuration, cell type, and microenvironmental context (41–43). Notch-related signaling has also been implicated in VM in glioblastoma and breast cancer, mainly by maintaining a stem-like state and reducing the epithelial cohesion required for proper lumen formation in three dimensions (41). In NSCLC, however, VM depends more strongly on the HIF-1α–EGFR axis than on the Notch-driven mechanisms that dominate in melanoma (19). 

Taken together, the bibliometric and experimental literature suggests that TGF-β, hypoxia/HIF-1α, and Notch-related signaling may represent partially overlapping regulatory contexts rather than a single shared causal hub. These signals can influence endothelial plasticity and tumor-cell plasticity through partly distinct mechanisms, while their convergence within the tumor microenvironment provides a plausible basis for interaction between EndMT and VM. The available evidence is currently stronger for parallel or indirect effects than for direct reciprocal regulation. Accordingly, the relationship between the two processes should be considered a testable hypothesis rather than an established bidirectional mechanism.

### 4.2 The stromal interface: EndMT-derived CAFs, ECM remodeling, and VM permissiveness

A second point of mechanistic convergence emerges in the stromal microenvironment. The bibliometric patterns point directly to this interface. In the EndMT co-occurrence network (**Figure 9A**), the green peripheral cluster sits between the cardiovascular disease core (red) and the molecular biology axis (blue). It brings together terms such as extracellular matrix, myofibroblast, vasculotropin, and phenotype. Its placement at the boundary of major clusters visually marks it as the zone where EndMT research aligns most strongly with tumor biology and, by extension, VM. The VM network (**Figure 9B**) shows a similar structure. Stromal and matrix-related terms appear in bridging positions that link the central oncology cluster to the experimental mechanism cluster, suggesting that VM researchers also treat the microenvironment as a connective layer between cellular plasticity and tumor behavior. The thematic maps strengthen this interpretation. In the EndMT map (**Figure 11A**), ECM remodeling and cancer-associated fibroblasts fall in the basic-theme quadrant — high centrality but low internal density. This pattern characterizes topics that matter to many parts of the field yet have not matured into independent research hubs. It is the expected profile for concepts that sit at the structural center of EndMT’s relationship with tumor biology but whose mechanistic detailing remains incomplete. Across analyses, the same factors recur. ECM remodeling, cancer-associated fibroblasts (CAFs), and matrix metalloproteinases (MMPs) consistently occupy central positions within the green cluster, which acts as the interface between EndMT’s cardiovascular foundations and which acts as an interface between the cardiovascular origins of EndMT research and the tumor-focused literature on VM. The recurrence of ECM remodeling, CAFs, and MMP-related terms across these analyses suggests that the stromal microenvironment may represent an important interface between the two fields. EndMT-associated stromal cells may contribute to matrix remodeling and protease activity, processes that have also been implicated in the formation and maintenance of VM structures.

Lineage-tracing studies provide evidence that endothelial-lineage cells can contribute to CAF-like populations in selected tumor contexts, including melanoma, pancreatic, and colorectal cancers. Single-cell analyses have further identified EndMT-associated stromal populations retaining partial endothelial features (9, 37). However, these findings do not establish that all such populations represent fully converted mesenchymal cells, and marker expression alone cannot distinguish complete lineage conversion from partial or hybrid states (24, 37, 44, 45). Importantly, the presence of residual endothelial features does not preclude these populations from acquiring functional stromal or CAF-like properties, including the capacity to remodel the extracellular matrix (9, 37, 38). EndMT-derived CAFs participate in an MMP–CAF–ECM axis; studies of CAF populations more broadly have shown that MMP-1, MMP-3, and MMP-10 are upregulated in a largely p53-dependent manner (46, 47), consistent with the well-established role of wild-type p53 as a transcriptional repressor of matrix metalloproteinase expression — a pattern that may extend to their EndMT-derived counterparts. MMP-2 and MT1-MMP (MMP-14) further drive matrix remodeling and basement membrane disruption at the tumor–stroma interface (9, 15). 

Beyond ECM composition, EndMT-derived CAFs also create a chemokine environment that influences tumor cell behavior. TGF-β1, CXCL12, and HGF secreted by these stromal cells promote EMT in neighboring carcinoma cells (37, 38). Because EMT and VM share several key transcriptional regulators — including Snail, Twist, and members of the ZEB family — it is reasonable to hypothesize that CAF-driven paracrine induction of EMT could lower the threshold for VM activation in neighboring tumor cells. However, this remains a testable mechanistic hypothesis: direct experimental evidence linking the secretome of EndMT-associated stromal cells to VM initiation in co-culture or *in vivo* models remains limited.

In addition, CAF-derived laminin interacts with integrin α6β4 on tumor cells, triggering migration (48). In melanoma and breast cancer models, integrin α6β4 signaling partially converges with pathways involved in VM tube formation; however, a direct causal role for this interaction in VM, particularly within EndMT-rich stroma, has not yet been formally demonstrated.

Taken together, these observations support a testable model in which EndMT-associated stromal remodeling may increase the permissiveness of the tumor microenvironment for VM. This proposed relationship is more consistent with an indirect microenvironmental interaction than with direct EndMT-to-VM conversion.

### 4.3 Partial phenotypes and hybrid transition states as a mechanistic bridge

A third, conceptually distinct point of intersection between EndMT and VM comes from the growing recognition that both processes rely on graded and reversible phenotypic shifts rather than clean binary transitions. The bibliometric signals for this axis are especially telling because they appear independently across three analytical layers. The first signal comes from the citation burst analysis of the EndMT corpus (**Figure 10A**). Here, the most recent burst wave shows tumor necrosis factor (5.95, 2022–2025) and differential gene expression (6.69, 2023–2025) as the leading active terms, alongside shear stress (5.27, 2020–2023) and flow (5.65, 2021–2022). Taken together, these bursts point to a broad shift in attention: researchers are looking less at EndMT as a fixed endpoint and more at its dynamic, context-dependent regulation under mechanical, inflammatory, and transcriptomic conditions. A second, parallel signal appears in the VM burst analysis (**Figure 10B**). Resistance (13.59, 2019–2025) and tumor microenvironment (12.71, 2020–2025) emerge as dominant frontier keywords, while vascular mimicry itself shows the second strongest burst in the corpus (23.01, 2020–2025), reflecting increasing research attention to plasticity-driven and microenvironment-responsive cellular states in relation to VM competence. This mirrors the shift under way in EndMT research, even though the two communities have largely developed their frameworks independently. The third line of evidence comes from the thematic maps (**Figure 11**). Vocabulary related to partial or hybrid transition states is located in the emerging-theme quadrants of both corpora. This placement indicates that these ideas are gaining momentum but have not yet matured into stable, high-density research clusters. The parallel positioning of these themes suggests a potential area of convergence between the two research fields, although the biological significance of this bibliometric similarity remains to be established. Together, these temporal and structural trends suggest that both research communities are increasingly focusing on dynamic and context-dependent cellular states rather than exclusively on fully differentiated endpoints: hybrid transition states — rather than fully differentiated endpoints — are the configurations that matter most in the tumor microenvironment.

The concept of partial or hybrid EndMT has received substantial experimental support. *In vivo*, tumor-derived endothelial cell populations include cells that co-express both endothelial markers (CD31/VE-cadherin) and mesenchymal markers such as FSP-1, α-SMA, and vimentin (37). A reporter-based EndMT visualization system (EMRECs), designed to track the sequential stages of EndMT, has identified CD40 as a surface marker for partial EndMT intermediates within the tumor microenvironment, confirming that these hybrid cells form a reproducible, functionally distinct population rather than representing mere transitional noise (44). Partial EndMT is transient and reversible, playing a role in both developmental angiogenesis and pathological vascular remodeling. The process may be regulated by a balance between pro-EndMT signals (such as TGF-β, hypoxia, and Notch) and endothelial maintenance signals such as bFGF (24, 45). 

The analogy between partial EndMT and partial EMT in tumor cells is both precise and mechanistically instructive. Hybrid epithelial/mesenchymal (E/M) cells — which co-express both epithelial and mesenchymal markers — exhibit greater tumorigenic properties than either purely epithelial or purely mesenchymal cells. These properties include cancer stem cell (CSC) traits, increased metastatic capacity, collective migration, and therapy resistance (11, 49). This hybrid state is especially prominent at the tumor invasive front and in circulating tumor cells, and its maintenance relies on phenotypic stability factors (PSFs) that establish a bistable or multistable landscape between epithelial and mesenchymal states (11, 50). In terms of VM, hybrid E/M states have been associated with greater VM competence in several tumor models: fully mesenchymal or fully epithelial cells are less likely to form VM channels than hybrid cells, possibly because VM requires both the mesenchymal program (driven by Snail and VE-cadherin/EphA2 expression) and the cohesive, channel-forming capacity of partially epithelial cells (18, 35). 

This framework raises the possibility that partial EndMT intermediates may influence VM indirectly through interactions with neighboring tumor cells and the surrounding microenvironment. Whether partial EndMT cells themselves can acquire VM-forming capacity remains unknown. In particular, it remains unclear whether such cells could directly form VM-like structures or instead influence tumor cells through contact-dependent or paracrine mechanisms. This question has not been directly addressed in the current literature, which includes only five publications examining EndMT and VM together in the tumor context. Approaches such as deploying the EMREC reporter system in co-culture with VM-competent tumor cell lines, or conducting spatial transcriptomic profiling of VM-positive tumor regions for partial EndMT markers, could provide experimentally testable approaches to distinguish these possibilities.

### 4.4 EndMT and VM in the context of anti-angiogenic therapy

The potential relevance of EndMT and VM to anti-angiogenic therapy resistance should be considered within the broader heterogeneity of tumor vascular adaptation. Anti-angiogenic agents such as bevacizumab and sunitinib can produce different vascular responses depending on tumor type, therapeutic agent, dose, treatment duration, and baseline vascular state (7, 10–12). These responses may include transient vascular normalization, vessel pruning, persistent vascular dysfunction, hypoxia, activation of alternative pro-angiogenic pathways, vessel co-option, and changes in endothelial or tumor-cell states (10–12, 51). 

Experimental studies have shown that VEGF-targeted treatment can increase hypoxia-associated VM in selected tumor models. For example, short-term bevacizumab treatment increased HIF-1α expression and VM channel density in an ovarian cancer model (13). Sunitinib has also been reported to promote VM-related tumor-cell plasticity through hypoxia-associated mechanisms (14). These findings support the possibility that treatment-induced vascular stress may favor VM in particular tumor contexts. However, they should not be generalized to all anti-angiogenic therapies or tumor types (8, 11). 

Importantly, the same treatment-induced vascular stress may also affect endothelial plasticity. Hypoxia can promote EndMT-associated changes through HIF-1α and related pathways (52). Thus, EndMT and VM may emerge as distinct adaptive responses under overlapping conditions of vascular stress, rather than as sequential stages of a single resistance pathway (15, 16, 32). Current evidence does not establish a universal sequence in which VEGF inhibition causes EndMT followed by replacement of conventional endothelial vessels by VM. Nor should VM be interpreted simply as a functional substitute for endothelial vasculature.

A more cautious interpretation is that anti-angiogenic treatment may alter the tumor microenvironment in ways that independently or jointly promote several adaptive phenotypes, including EndMT-associated stromal remodeling and VM (11, 13, 14, 16). Determining whether these responses interact directly, indirectly through the stromal compartment, or primarily coexist under particular treatment conditions will require longitudinal, lineage-resolved, and spatially resolved experimental studies.

## 5 Discussion

### 5.1 Interpreting the dual-topic research landscape: biological convergence and academic separation

To our knowledge, this is the first bibliometric study to examine endothelial-to-mesenchymal transition (EndMT) and vasculogenic mimicry (VM) side by side within tumor biology. Both processes represent forms of cellular plasticity within the tumor vascular microenvironment, but they arise from different cellular origins and involve distinct phenotypic programs. EndMT describes a spectrum of endothelial phenotypic change toward mesenchymal-like states, whereas VM involves tumor cells acquiring vessel-like properties and organizing into non-endothelial vascular structures (4–6, 24, 26). Their distinct biological definitions notwithstanding, both processes have been associated with hypoxia, extracellular-matrix remodeling, cellular plasticity, and therapeutic stress in specific tumor contexts (8, 13–16, 39). 

One of the clearest findings from our bibliometric analysis is the marked academic separation between these two research areas. Although both literatures address aspects of tumor vascular plasticity and share several recurring biological themes, only five publications explicitly addressed EndMT and VM together within the tumor context. These five records represented approximately 0.105% of the 4,746 unique publications in the combined dataset. This limited overlap indicates that the two fields have developed largely in parallel rather than as an integrated research area.

The separation appears to be related, at least in part, to their different disciplinary origins. EndMT research developed largely from cardiovascular biology, fibrosis, developmental vascular biology, and vascular remodeling (4, 23, 25, 26), whereas VM research emerged primarily from oncology and tumor pathology (5, 6). These origins remain visible in journal distributions, institutional contributions, and collaboration patterns identified in our analysis. EndMT research continues to be closely connected to vascular and translational biology, whereas VM research is more strongly embedded in studies of tumor progression, invasion, metastasis, and therapeutic resistance (4, 5, 19, 20). Consequently, related concepts are often investigated using different terminology and conceptual frameworks, which may contribute to the limited cross-citation between the two fields.

Importantly, the small number of studies addressing both processes should not be interpreted as evidence that they are biologically unrelated. Across the bibliometric analyses, the two literatures showed partially overlapping attention to the tumor microenvironment, cellular plasticity, extracellular-matrix remodeling, EMT-related programs, and therapeutic resistance. These patterns identify a shared conceptual space in which interactions between endothelial and tumor-cell plasticity may occur. However, bibliometric convergence does not establish a direct biological relationship. Rather, it provides a rationale for examining whether the two processes influence one another directly, interact indirectly through the stromal microenvironment, or simply arise in parallel under similar environmental pressures.

Viewed in this way, the limited cross-literature overlap represents not only a gap in scholarly integration but also an opportunity for experimental investigation. The present study therefore does not propose that EndMT and VM constitute a single biological program. Instead, it identifies areas of thematic and mechanistic convergence that may provide a basis for future studies testing their potential interactions within defined tumor contexts.

### 5.2 Emerging research frontiers revealed by bibliometric signals

Temporal trends in the literature further support the emergence of partially overlapping research priorities. In the VM field, recent keywords—including tumor microenvironment, epithelial–mesenchymal transition, resistance, and drug resistance—indicate increasing attention to tumor-cell plasticity, microenvironmental regulation, and therapeutic adaptation (5, 11, 35, 50).  A similar broadening is evident in EndMT research, where recent activity increasingly addresses endothelial dysfunction, mesenchymal reprogramming, stromal remodeling, inflammatory signaling, and mechanical or hemodynamic regulation (4, 16, 24–26, 33, 34). 

The keyword co-occurrence networks provide additional evidence for this thematic convergence. In the EndMT network, extracellular-matrix remodeling, myofibroblast-related phenotypes, and tumor-associated stromal features occupy positions connecting vascular biology with oncological applications (9, 15, 37, 38). In the VM network, EMT-related plasticity and microenvironmental terms similarly connect core VM concepts with broader malignant phenotypes (5, 35, 49, 50). These bridging themes may therefore provide useful entry points for further cross-disciplinary investigation of the two literatures.

An important implication of these findings is that bibliometric analysis can identify areas of conceptual convergence even when direct collaboration or cross-citation remains limited. In the present study, both EndMT and VM research increasingly address matrix remodeling, cellular plasticity, microenvironmental adaptation, and therapeutic resistance (5, 8, 9, 11, 15, 35, 37). These shared priorities may provide useful entry points for cross-disciplinary investigation. However, this convergence should be interpreted as a research opportunity rather than evidence of an established common mechanism.

### 5.3 Methodological contributions and limitations

This study also makes several methodological contributions. By integrating two major bibliographic databases and applying a unified deduplication and screening workflow, we constructed comparable literature datasets for EndMT and VM covering the same time period. This approach improves literature coverage and helps reduce potential bias associated with reliance on a single database source. In addition, the parallel analytical framework enables two closely related but historically separate research areas to be compared directly across multiple dimensions, including research output, citation patterns, journal distribution, national and institutional contributions, and thematic development over time. By explicitly identifying cross-topic publications, the study also provides a quantitative indicator of the degree of academic separation between the two fields and highlights potential gaps that may be less apparent when the two literatures are examined separately.

Several limitations should also be considered. Bibliometric analysis primarily reflects patterns of publication and citation and does not directly measure the biological importance of specific mechanisms. Highly cited or frequently studied topics are not necessarily the most critical biological questions, while less prominent research directions may still hold substantial importance. In addition, keyword-based thematic mapping depends heavily on author terminology and database indexing practices. Different expressions or terminological variants may distribute conceptually related studies across separate thematic clusters. For this reason, the number of cross-literature articles identified in this study may underestimate the true extent of research addressing EndMT and VM simultaneously.

Another limitation concerns the temporal scope of the dataset. The quantitative analysis begins in 2005 and therefore does not include some of the earliest foundational VM studies published between 1999 and 2004, which retain important conceptual influence in the field. This limitation primarily affects the historical interpretation of VM development rather than the comparability of the two datasets during the selected study period. Finally, the mechanistic integration framework proposed here remains partly hypothetical. Although it is grounded in existing experimental observations from both research areas, several proposed connections still require direct validation. For example, it remains unclear whether EndMT-derived stromal cells can promote VM formation, or whether transitional endothelial cell states exist that directly participate in the formation of non-endothelial vascular structures.

### 5.4 Directions for future research

Integrating the bibliometric findings with the mechanistic synthesis, this study proposes several future research directions. Spatially resolved tumor studies represent an important starting point. It remains necessary to determine whether EndMT and VM co-occur within the same microanatomical regions, particularly at hypoxic invasion fronts or in areas subjected to sustained therapeutic pressure. Advances in multiplex immunohistochemistry, spatial transcriptomics, and high-resolution tissue imaging now make this question experimentally approachable. If tumors displaying evidence of both processes represent a distinct biological subtype characterized by unusually high vascular plasticity, such a distinction could have implications for prognosis and treatment stratification (15, 17, 25). 

A second area for future investigation concerns the stromal interface and the directionality of potential interactions between the two processes. Our mechanistic analysis suggests a testable possibility in which EndMT-associated stromal remodeling may alter the extracellular-matrix and paracrine environment in ways that increase the permissiveness of neighboring tumor cells for VM (9, 15, 27, 37). Candidate components include matrix remodeling, protease activity, and stromal factors such as TGF-β and CXCL12/SDF-1 (9, 15, 27, 37, 38). However, this proposed relationship remains hypothetical, and the current literature does not establish a direct causal pathway from EndMT to VM. This possibility can be investigated using co-culture systems, conditioned-medium experiments, organoid models, lineage tracing, and spatially resolved approaches. Such studies could determine whether EndMT-associated cells directly enhance VM competence in neighboring tumor cells, whether the effect is mediated by specific extracellular-matrix or paracrine signals, or whether the two phenotypes simply coexist under shared microenvironmental conditions.

The relationship between EndMT and VM may also be relevant to resistance against anti-angiogenic therapy (4, 8, 11). Tumors exposed to VEGF-targeted treatments may adapt through broader vascular plasticity programs that involve not only alternative angiogenic pathways but also stromal reprogramming and tumor cell–driven vascular mimicry (13, 14). Prospective studies could examine whether combined molecular or spatial signatures of EndMT-associated remodeling and VM are associated with poor therapeutic response, shorter progression-free survival, or increased metastatic risk (20, 22). Such studies would help determine whether the coexistence of these phenotypes identifies a clinically meaningful pattern of vascular plasticity.

Overall, our findings support a broader view of tumor vascular plasticity in which EndMT and VM represent distinct but potentially interacting forms of cellular adaptation (4, 5, 15, 35). EndMT involves phenotypic plasticity of endothelial cells, whereas VM reflects the acquisition of vessel-like properties by tumor cells (4, 5). Their potential relationship may therefore arise through shared environmental pressures, partially overlapping signaling pathways, or interactions mediated by the stromal microenvironment rather than through a single sequential pathway (15, 27, 35, 37). Importantly, the present study does not establish a bidirectional causal relationship between EndMT and VM. The biological relationship may vary across tumor types, anatomical niches, and treatment conditions.

Future studies combining lineage tracing, spatial profiling, and functional co-culture or organoid models will be needed to determine whether EndMT and VM directly influence one another, interact through stromal intermediates, or primarily represent distinct adaptive responses to shared microenvironmental pressures.

### 5.5 Conclusions

This study provides, to our knowledge, the first comparative bibliometric analysis of EndMT and VM within tumor biology. Using a combined corpus of 4,746 unique publications retrieved from Web of Science and Scopus, we found that these two research areas have developed largely independently despite partially overlapping interests in tumor vascular plasticity, hypoxia, cellular plasticity, extracellular-matrix remodeling, and therapeutic resistance (4, 5, 11, 35). Only five publications explicitly addressed both EndMT and VM within the tumor context, representing approximately 0.105% of the combined unique corpus.

Bibliometric analyses further showed that the two literatures are increasingly converging on related research priorities, particularly the tumor microenvironment, EMT-related plasticity, stromal remodeling, and treatment resistance (5, 9, 11, 35, 37). These findings indicate a growing conceptual convergence between the fields, but they do not establish a shared causal mechanism.

Considered alongside relevant experimental literature, the bibliometric findings point to three areas for future investigation: partially overlapping signaling contexts involving TGF-β, hypoxia/HIF-1α, and Notch-related pathways (15, 26, 32, 39); the potential role of EndMT-associated stromal remodeling in shaping VM-permissive microenvironments (9, 15, 27, 37); and the contribution of partial or hybrid cellular states to tumor vascular plasticity (24, 44, 45, 49). Across these areas, current evidence supports parallel or indirect interactions more strongly than direct reciprocal regulation.

The framework proposed here should therefore be regarded as a hypothesis-generating model rather than an established mechanistic pathway. Future lineage-resolved, spatially resolved, and functional studies will be needed to determine whether EndMT and VM directly influence one another, interact through stromal intermediates, or primarily represent distinct adaptive responses to shared tumor microenvironmental pressures. Clarifying these relationships may provide a more integrated understanding of tumor vascular plasticity and identify broader therapeutic opportunities beyond conventional angiogenic mechanisms (8, 10–13). 

## Data Availability

The datasets presented in this study can be found in online repositories. The names of the repository/repositories and accession number(s) can be found in the article.

## References

[1] Beck AW Chen H Ren B . Arteriolar angiogenesis: implications in anti-angiogenic immunotherapy for pancreatic cancers. Cytokine Growth Fact Rev. (2025) 86:96–107. doi: 10.1016/j.cytogfr.2025.10.002 41106113 PMC12794125

[2] Hanahan D Weinberg RA . Hallmarks of cancer: the next generation. Cell. (2011) 144:646–74. doi: 10.1016/j.cell.2011.02.013 21376230

[3] Carmeliet P Jain RK . Molecular mechanisms and clinical applications of angiogenesis. Nature. (2011) 473:298–307. doi: 10.1038/nature10144 21593862 PMC4049445

[4] Xu Y Kovacic JC . Endothelial to mesenchymal transition in health and disease. Annu Rev Physiol. (2023) 85:245–67. doi: 10.1146/annurev-physiol-032222-080806 36266259

[5] Luo Q Wang J Zhao W Peng Z Liu X Li B . Vasculogenic mimicry in carcinogenesis and clinical applications. J Hematol Oncol. (2020) 13:19. doi: 10.1186/s13045-020-00858-6 32169087 PMC7071697

[6] Maniotis AJ Folberg R Hess A Seftor EA Gardner LM Pe'er J . Vascular channel formation by human melanoma cells *in vivo* and *in vitro*: vasculogenic mimicry. Am J Pathol. (1999) 155:739–52. doi: 10.1016/s0002-9440(10)65173-5 10487832 PMC1866899

[7] Liu ZL Chen HH Zheng LL Sun LP Shi L . Angiogenic signaling pathways and anti-angiogenic therapy for cancer. Signal Transd Targ Ther. (2023) 8:198. doi: 10.1038/s41392-023-01460-1 37169756 PMC10175505

[8] Haibe Y Kreidieh M El Hajj H Khalifeh I Mukherji D Temraz S . Resistance mechanisms to anti-angiogenic therapies in cancer. Front Oncol. (2020) 10:221. doi: 10.3389/fonc.2020.00221 32175278 PMC7056882

[9] Han J Kim EG Kim BY Soung NK . Cancer-associated fibroblasts arising from endothelial-to-mesenchymal transition: induction factors, functional roles, and transcriptomic evidence. Biology. (2025) 14. doi: 10.3390/biology14101403 41154806 PMC12561803

[10] Ribatti D Solimando AG Pezzella F . The anti-VEGF(R) drug discovery legacy: improving attrition rates by breaking the vicious cycle of angiogenesis in cancer. Cancers. (2021) 13. doi: 10.3390/cancers13143433 34298648 PMC8304542

[11] Huang M Lin Y Wang C Deng L Chen M Assaraf YG . New insights into antiangiogenic therapy resistance in cancer: mechanisms and therapeutic aspects. Drug Resist Updates: Rev Commentaries Antimicrob Anticancer Chemother. (2022) 64:100849. doi: 10.1016/j.drup.2022.100849 35842983

[12] Yang S Fang Y Ma Y Wang F Wang Y Jia J . Angiogenesis and targeted therapy in the tumour microenvironment: from basic to clinical practice. Clin Transl Med. (2025) 15:e70313. doi: 10.1002/ctm2.70313 40268524 PMC12017902

[13] Xu Y Li Q Li XY Yang QY Xu WW Liu GL . Short-term anti-vascular endothelial growth factor treatment elicits vasculogenic mimicry formation of tumors to accelerate metastasis. J Exp Clin Cancer Res: CR. (2012) 31:16. doi: 10.1186/1756-9966-31-16 22357313 PMC3310846

[14] Zhang D Sun B Zhao X Ma Y Ji R Gu Q . Twist1 expression induced by sunitinib accelerates tumor cell vasculogenic mimicry by increasing the population of CD133+ cells in triple-negative breast cancer. Mol Cancer. (2014) 13:207. doi: 10.1186/1476-4598-13-207 25200065 PMC4168051

[15] Du F Li J Zhong X Zhang Z Zhao Y . Endothelial-to-mesenchymal transition in the tumor microenvironment: roles of transforming growth factor-β and matrix metalloproteins. Heliyon. (2024) 10:e40118. doi: 10.1016/j.heliyon.2024.e40118 39568849 PMC11577214

[16] Choi KJ Nam JK Kim JH Choi SH Lee YJ . Endothelial-to-mesenchymal transition in anticancer therapy and normal tissue damage. Exp Mol Med. (2020) 52:781–92. doi: 10.1038/s12276-020-0439-4 32467609 PMC7272420

[17] Ma X Geng Z Wang S Yu Z Liu T Guan S . The driving mechanism and targeting value of mimicry between vascular endothelial cells and tumor cells in tumor progression. Biomed Pharmacother = Biomed Pharmacother. (2023) 165:115029. doi: 10.1016/j.biopha.2023.115029 37343434

[18] Tang H Chen L Liu X Zeng S Tan H Chen G . Pan-cancer dissection of vasculogenic mimicry characteristic to provide potential therapeutic targets. Front Pharmacol. (2024) 15:1346719. doi: 10.3390/ijms241813770 38694917 PMC11061449

[19] Shapkina V Shindyapin V Burlov N Prosekina E Artemyeva A . Vasculogenic mimicry in non-small cell lung cancer: a systematic review. Front Oncol. (2025) 15:1481726. doi: 10.3389/fonc.2025.1481726 40786517 PMC12331492

[20] Yang JP Liao YD Mai DM Xie P Qiang YY Zheng LS . Tumor vasculogenic mimicry predicts poor prognosis in cancer patients: a meta-analysis. Angiogenesis. (2016) 19:191–200. doi: 10.1007/s10456-016-9500-2 26899730

[21] Cao Z Bao M Miele L Sarkar FH Wang Z Zhou Q . Tumour vasculogenic mimicry is associated with poor prognosis of human cancer patients: a systemic review and meta-analysis. Eur J Cancer (Oxford Engl 1990). (2013) 49:3914–23. doi: 10.1016/j.ejca.2013.07.148 23992642

[22] Chen J Wang Y Wu M Yu K Liu J Chang J . Vasculogenic mimicry triggers early recidivation and resistance to adjuvant therapy in esophageal cancer. BMC Cancer. (2024) 24:1132. doi: 10.1186/s12885-024-12903-5 39261780 PMC11389244

[23] Zeisberg EM Tarnavski O Zeisberg M Dorfman AL McMullen JR Gustafsson E . Endothelial-to-mesenchymal transition contributes to cardiac fibrosis. Nat Med. (2007) 13:952–61. doi: 10.1038/nm1613 17660828

[24] Fang JS Hultgren NW Hughes CCW . Regulation of partial and reversible endothelial-to-mesenchymal transition in angiogenesis. Front Cell Dev Biol. (2021) 9:702021. doi: 10.3389/fcell.2021.702021 34692672 PMC8529039

[25] Singh A Bhatt KS Nguyen HC Frisbee JC Singh KK . Endothelial-to-mesenchymal transition in cardiovascular pathophysiology. Int J Mol Sci. (2024) 25. doi: 10.3390/ijms25116180 PMC11173124 38892367

[26] Ma J Sanchez-Duffhues G Goumans MJ Ten Dijke P . TGF-β-induced endothelial to mesenchymal transition in disease and tissue engineering. Front Cell Dev Biol. (2020) 8:260. doi: 10.3389/fcell.2020.00260 32373613 PMC7187792

[27] Yang J Lu Y Lin YY Zheng ZY Fang JH He S . Vascular mimicry formation is promoted by paracrine TGF-β and SDF1 of cancer-associated fibroblasts and inhibited by miR-101 in hepatocellular carcinoma. Cancer Lett. (2016) 383:18–27. doi: 10.1016/j.canlet.2016.09.012 27693460

[28] Morales-Guadarrama G Méndez-Pérez EA García-Quiroz J Avila E Ibarra-Sánchez MJ Esparza-López J . The inhibition of the FGFR/PI3K/Akt axis by AZD4547 disrupts the proangiogenic microenvironment and vasculogenic mimicry arising from the interplay between endothelial and triple-negative breast cancer cells. Int J Mol Sci. (2023) 24. doi: 10.20944/preprints202307.1203.v1 PMC10531243 37762073

[29] Hernández-Camarero P Toledo B Diaz-Ruano AB González-Titos A García-Ortega MB Perán M . What is the impact of endothelial-to-mesenchymal transition in solid tumours: a qualitative systematic review and quantitative meta-analysis. Int J Biol Sci. (2025) 21:2155–78. doi: 10.7150/ijbs.107045 40083695 PMC11900810

[30] Aria M Cuccurullo C . bibliometrix: an R-tool for comprehensive science mapping analysis. J Informetr. (2017) 11:959–75. doi: 10.1016/j.joi.2017.08.007

[31] van Eck NJ Waltman L . Software survey: VOSviewer, a computer program for bibliometric mapping. Scientometrics. (2010) 84:523–38. doi: 10.1007/s11192-009-0146-3 20585380 PMC2883932

[32] Ma J van der Zon G Gonçalves M van Dinther M Thorikay M Sanchez-Duffhues G . TGF-β-induced endothelial to mesenchymal transition is determined by a balance between SNAIL and ID factors. Front Cell Dev Biol. (2021) 9:616610. doi: 10.3389/fcell.2021.616610 33644053 PMC7907445

[33] Shen Z Yang S Zhang Q Liu Q Wang H Tao J . Research advances in epigenetic modifications and post-translational modifications in endothelial-mesenchymal transition. Epigenet Chromatin. (2025) 18:80. doi: 10.1186/s13072-025-00649-8 41382207 PMC12699931

[34] Jiang YZ Jiménez JM Ou K McCormick ME Zhang LD Davies PF . Hemodynamic disturbed flow induces differential DNA methylation of endothelial Kruppel-like factor 4 promoter *in vitro* and *in vivo*. Circ Res. (2014) 115:32–43. doi: 10.1161/circresaha.115.303883 24755985 PMC4065854

[35] Serratì S Raho L De Giosa G Porcelli L Di Fonte R Fasano R . Unraveling vascular mechanisms in melanoma: roles of angiogenesis and vasculogenic mimicry in tumor progression and therapeutic resistance. Cancer Biol Med. (2025) 22:1327–52. doi: 10.20892/j.issn.2095-3941.2025.0048 41251262 PMC12624814

[36] Delgado-Bellido D Oliver FJ Vargas Padilla MV Lobo-Selma L Chacón-Barrado A Díaz-Martin J . VE-cadherin in cancer-associated angiogenesis: a deceptive strategy of blood vessel formation. Int J Mol Sci. (2023) 24. doi: 10.3390/ijms24119343 37298296 PMC10253758

[37] Giordanengo L Proment A Botta V Picca F Munir HMW Tao J . Shifting shapes: the endothelial-to-mesenchymal transition as a driver for cancer progression. Int J Mol Sci. (2025) 26. doi: 10.3390/ijms26136353 40650130 PMC12250086

[38] Singh S Singh AP Mitra R . Cancer-associated fibroblasts: major co-conspirators in tumor development. Cancers. (2024) 16. doi: 10.3390/cancers16010211 38201638 PMC10778099

[39] Saha D Mitra D Alam N Sen S Mustafi SM Majumder PK . Lupeol and paclitaxel cooperate in hindering hypoxia induced vasculogenic mimicry via suppression of HIF-1α-EphA2-laminin-5γ2 network in human oral cancer. J Cell Commun Signaling. (2023) 17:591–608. doi: 10.1007/s12079-022-00693-z 36063341 PMC10409936

[40] Zhao N Sun BC Sun T Ma YM Zhao XL Liu ZY . Hypoxia-induced vasculogenic mimicry formation via VE-cadherin regulation by Bcl-2. Med Oncol (Northwood London England). (2012) 29:3599–607. doi: 10.1007/s12032-012-0245-5 22562824

[41] Akil A Gutiérrez-García AK Guenter R Rose JB Beck AW Chen H . Notch signaling in vascular endothelial cells, angiogenesis, and tumor progression: an update and prospective. Front Cell Dev Biol. (2021) 9:642352. doi: 10.3389/fcell.2021.642352 33681228 PMC7928398

[42] Hellström M Phng LK Hofmann JJ Wallgard E Coultas L Lindblom P . Dll4 signalling through Notch1 regulates formation of tip cells during angiogenesis. Nature. (2007) 445:776–82. doi: 10.1038/nature05571 17259973

[43] Benedito R Roca C Sörensen I Adams S Gossler A Fruttiger M . The notch ligands Dll4 and Jagged1 have opposing effects on angiogenesis. Cell. (2009) 137:1124–35. doi: 10.1016/j.cell.2009.03.025 19524514

[44] Takahashi K Kobayashi M Katsumata H Tokizaki S Anzai T Ikeda Y . CD40 is expressed in the subsets of endothelial cells undergoing partial endothelial-mesenchymal transition in tumor microenvironment. Cancer Sci. (2024) 115:490–506. doi: 10.1111/cas.16045 38111334 PMC10859613

[45] Xiao L Kim DJ Davis CL McCann JV Dunleavey JM Vanderlinden AK . Tumor endothelial cells with distinct patterns of TGFβ-driven endothelial-to-mesenchymal transition. Cancer Res. (2015) 75:1244–54. doi: 10.1158/0008-5472.can-14-1616 25634211 PMC4383705

[46] Arandkar S Furth N Elisha Y Nataraj NB van der Kuip H Yarden Y . Altered p53 functionality in cancer-associated fibroblasts contributes to their cancer-supporting features. PNAS. (2018) 115:6410–15. doi: 10.1073/pnas.1719076115 29866855 PMC6016816

[47] Sarkar M Nguyen T Gundre E Ogunlusi O El-Sobky M Giri B . Cancer-associated fibroblasts: the chief architect in the tumor microenvironment. Front Cell Dev Biol. (2023) 11:1089068. doi: 10.3389/fcell.2023.1089068 36793444 PMC9923123

[48] Yu S Wang S Wang X Xu X . The axis of tumor-associated macrophages, extracellular matrix proteins, and cancer-associated fibroblasts in oncogenesis. Cancer Cell Int. (2024) 24:335. doi: 10.1186/s12935-024-03518-8 39375726 PMC11459962

[49] Subbalakshmi AR Kundnani D Biswas K Ghosh A Hanash SM Tripathi SC . NFATc acts as a non-canonical phenotypic stability factor for a hybrid epithelial/mesenchymal phenotype. Front Oncol. (2020) 10:553342. doi: 10.3389/fonc.2020.553342 33014880 PMC7506140

[50] Dongre A Weinberg RA . New insights into the mechanisms of epithelial-mesenchymal transition and implications for cancer. Nat Rev Mol Cell Biol. (2019) 20:69–84. doi: 10.1038/s41580-018-0080-4 30459476

[51] Zhao C Zeng Y Kang N Liu Y . A new perspective on antiangiogenic antibody drug resistance: Biomarkers, mechanisms, and strategies in Malignancies. Drug Dev Res. (2024) 85:e22257. doi: 10.1002/ddr.22257 39245913

[52] Xu X Tan X Tampe B Sanchez E Zeisberg M Zeisberg EM . Snail is a direct target of hypoxia-inducible factor 1α (HIF1α) in hypoxia-induced endothelial to mesenchymal transition of human coronary endothelial cells. J Biol Chem. (2015) 290:16653–64. doi: 10.1074/jbc.m115.636944 25971970 PMC4505417

